# Developmental patterns and individual differences in responding to social feedback: A longitudinal fMRI study from childhood to adolescence

**DOI:** 10.1016/j.dcn.2023.101264

**Published:** 2023-06-10

**Authors:** Simone Dobbelaar, Michelle Achterberg, Anna C.K. van Duijvenvoorde, Marinus H. van IJzendoorn, Eveline A. Crone

**Affiliations:** a Leiden Consortium on Individual Development, Leiden University, the Netherlands; b Developmental and Educational Psychology, Leiden University, the Netherlands; c Leiden Institute for Brain and Cognition, Leiden University, the Netherlands; d Department of Psychology, Education and Child Studies, Erasmus University Rotterdam, the Netherlands; e Research Department of Clinical, Education and Health Psychology, UCL, University of London, United Kingdom

**Keywords:** Social feedback, Parental sensitivity, Development, Childhood, Adolescence, FMRI

## Abstract

Learning to control behavior when receiving feedback underlies social adaptation in childhood and adolescence, and is potentially strengthened by environmental support factors, such as parents. This study examined the neural development of responding to social feedback from childhood to adolescence, and effects of parental sensitivity on this development. We studied these questions in a 3-wave longitudinal fMRI sample (ages 7–13 years, n = 512). We measured responses to feedback using the fMRI Social Network Aggression Task through noise blasts following peer feedback and associated neural activity, and parental sensitivity using observations of parent-child interactions during Etch-a-Sketch. Results revealed largest reductions in noise blasts following positive feedback between middle and late childhood and following negative feedback between late childhood and early adolescence. Additionally, brain-behavior associations between dorsolateral prefrontal cortex activation and noise blast durations became more differentiated across development. Parental sensitivity was only associated with noise blast duration following positive feedback in childhood, but not in adolescence. There was no relation between parental sensitivity and neural activity. Our findings contribute to our understanding of neural development and individual differences in responding to social feedback, and the role of parenting in supporting children’s adaption to social feedback.

## Highlights

• Three-wave longitudinal fMRI study on development of responses to social feedback from childhood to adolescence.
• Developmental trajectories differed for behavioral responses following positive, neutral and negative peer feedback.
• Inhibition of aggressive responses was more strongly associated with DLPFC activation during childhood than adolescence.
• Children with more sensitive parents showed more inhibition following positive feedback in middle childhood.

## 1 Introduction

The transition from childhood to adolescence is an important period for the development of social skills, as children increasingly engage in social interactions with classmates, friends and unknown peers. One important social skill is the ability to control behavioral responses when receiving feedback, for example in peer groups (e.g., Lansford et al., 2010). This social-affective ability has been studied in paradigms in which participants receive positive (i.e., acceptance) or negative (i.e., rejection) feedback from peers (Achterberg et al., 2018; Guyer et al., 2012). Typically, social rejection leads to heightened self-consciousness and negatively affects one’s self-image (Crone et al., 2020, Rodman et al., 2017).

Responses to social feedback can be experimentally investigated using the Social Network Aggression Task (Achterberg et al., 2016; based on Somerville et al., 2006). In this experimental task, participants receive positive, neutral or negative peer feedback which can lead to feelings of self-consciousness. Subsequently participants can respond by delivering a noise blast to the peer (see also Chester et al., 2014), which may help to protect self-image (Rodman et al., 2017). Prior work using this paradigm showed that between middle and late childhood, older children differentiate more in their responses to negative and positive social feedback than younger children (Achterberg et al., 2020, Dobbelaar et al., 2022), possibly reflecting more differentiation in behavioral control processes (Crone and Steinbeis, 2017, Zelazo and Carlson, 2012). This is consistent with research showing an increased differentiation in prosocial behavior towards liked and disliked peers in adolescence (Güroğlu et al., 2014). Developmental comparison studies have shown that reactive aggression, which is reflected in sending noise blasts, generally declines throughout development, although for some individuals aggression peaks in adolescence (Barker et al., 2006, Cui et al., 2016, Lickley and Sebastian, 2018). Taken together, even though prior studies examined the developmental patterns of experiencing social rejection (Sebastian et al., 2011), much less is known about the behavioral control development from childhood to adolescence after receiving social (i.e., negative, positive and neutral) feedback from peers.

The use of functional magnetic resonance imaging (fMRI) can contribute to our understanding of underlying processes related to this development, by focusing on neural activity during behavioral responses to social feedback. Prior studies using fMRI showed that activation in the dorsolateral prefrontal cortex (DLPFC) is associated with inhibitory and cognitive control processes in social and non-social contexts (Blasi et al., 2006, Durston et al., 2002, Ochsner et al., 2012). Specifically, in the Social Network Aggression Task, increased DLPFC activity during behavioral responses to positive (versus negative) social feedback may reflect intentional inhibition to deliver noise blasts following positive relative to negative feedback in childhood (Dobbelaar, Achterberg, van Drunen et al., 2022), but not in adulthood (van de Groep et al., 2021). In non-social inhibition tasks, the DLPFC was previously found to show decreased activation with increasing age (e.g., Ordaz et al., 2013). Possibly, the role of the DLPFC becomes more goal-directed across development (Crone and Dahl, 2012). However, the neural development of inhibition in social contexts from childhood to adolescence is not yet well understood. In this study, we examined how responses to social feedback and its neural correlates develop longitudinally, and which environmental factors contribute to this development, using a longitudinal fMRI sample of children who participated at ages 7–9-years, 9–11-years and 11–13-years.

An important question concerns whether developmental patterns are influenced by environmental support factors. Using behavioral genetic (twin) modelling, it was previously found that individual variation in responding to negative feedback and associated brain activation were mainly explained by environmental factors and/or measurement noise (Achterberg et al., 2018). The transition from childhood to adolescence is marked by an extension of the social world outside of the family context (Blakemore and Mills, 2014), but family support remains an important influence in children’s lives (e.g., Morris et al., 2017; Nickerson and Nagle, 2005). Parental sensitivity, the ability to notice, correctly interpret and adequately respond to a child’s signals (Ainsworth et al., 1974), has been shown to positively impact social development (Boeldt et al., 2012, Day and Padilla-Walker, 2009, Eisenberg et al., 2005, Neppl et al., 2020, O’Farrelly et al., 2021). That is, children with parents who were more sensitive to their needs were previously found to show more prosocial behavior, increased effortful control and less externalizing problems than children with less sensitive parents. There is not yet much research into the effects of parenting on brain development in childhood and adolescence (Tooley et al., 2021). One study reported that maternal criticism in late childhood and adolescence was associated with less activity in cognitive control and social cognitive areas (Lee et al., 2015). A second goal of this study was therefore to examine the influence of parental sensitivity on the development of responding to social feedback in the transition from childhood to early adolescence.

In the present preregistered study (Dobbelaar, Achterberg, van Duijvenvoorde et al., 2022), we used data of the longitudinal twin study of the Leiden Consortium on Individual Development (Crone et al., 2020, Euser et al., 2016). In this extensive longitudinal neuroimaging study, children were followed yearly from 7 to 13 years of age. Our first aim was to study the development of responding to social feedback from middle childhood to adolescence, both on a behavioral level (i.e., noise blast durations) as well as on a neural level (i.e., DLPFC activation during behavioral responses to social feedback). We expected that inhibitory control skills and associated DLPFC activity would increase towards adolescence.

Our second, preregistered aim was to test whether the development of parental sensitivity from middle to late childhood was associated with responses to social feedback in early adolescence. Our hypothesis stated that both parental sensitivity in middle childhood (intercept) as well as the development of parental sensitivity (slope) were related to increased inhibitory responses (i.e., shorter noise blast durations and increased DLPFC activity) in early adolescence.

An additional preregistered aim was to explore whether temperament moderated the association between parental sensitivity and responding to social feedback, to study which children might be most susceptible to parental influences. Therefore, in supplementary analyses, we explored whether the association between parental sensitivity and inhibitory responses would be stronger for children with more perceptual sensitivity compared to their peers (Ellis et al., 2011, Weeland et al., 2017).

## 2 Methods

### 2.1 Participants

This study was part of the longitudinal twin study of the Leiden Consortium on Individual Development (L-CID; Crone et al., 2020). Participants were recruited through municipality registries (Euser et al., 2016) and invitations for the middle childhood cohort were sent to families of same-sex twins born between 2006 and 2009 that lived in the Western municipalities of the Netherlands. Inclusion criteria were fluency in Dutch, normal or corrected to normal vision and no physical impairments that could hinder their performance on the behavioral tasks of the study. The study was approved by the Dutch Central Committee on Research Involving Human Subjects (CCMO, number NL50277.058.14) and written informed consent was obtained from both parents at the start of the study. At the fourth time point, participating children provided written informed consent as well. The data included in this study were collected in 2015–2016 (Time Point 1: T1; n = 512, mean age=7.95 ± 0.67 years), 2016–2017 (Time Point 2: T2; n = 494; mean age=8.94 ± 0.67 years), 2017–2018 (Time Point 3: T3; n = 456, mean age=9.98 ± 0.69 years) and 2019–2021 (Time Point 5: T5; n = 336; mean age=12.41 ± 0.76 years).

**Fig. 1:**
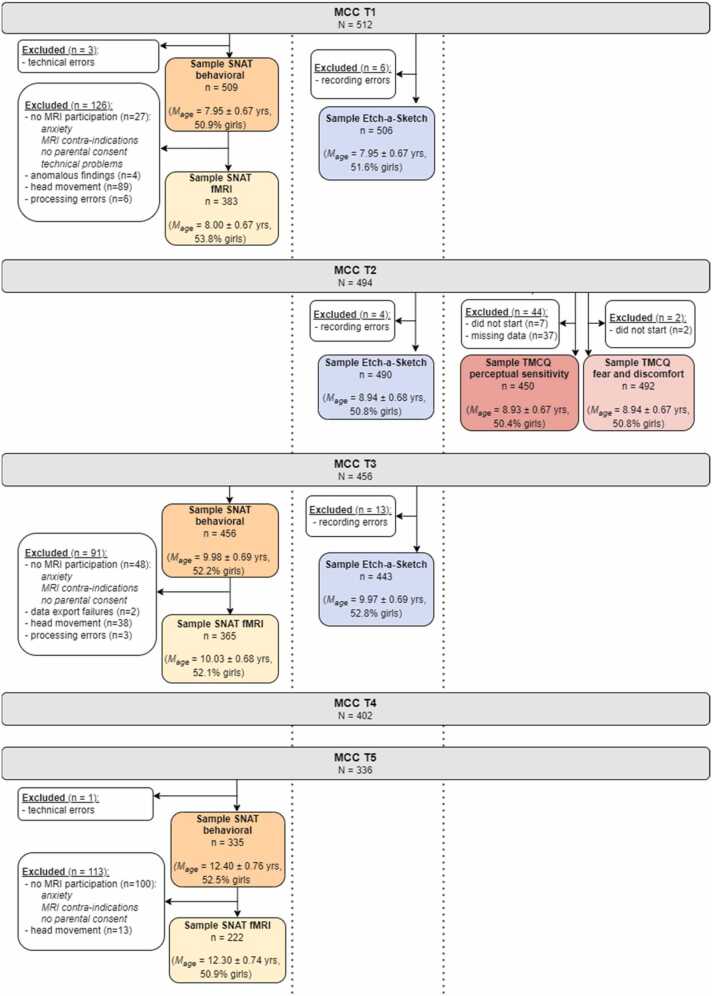
Flowchart of inclusion of participants for responses to feedback (SNAT and fMRI), parental sensitivity (Etch-a-Sketch) and temperament (TMCQ) variables at each time point.

An overview of demographic characteristics of the total sample at each time point is presented in Table 1 and Fig. 1. Measures on responses to feedback and neural activity (through fMRI) were collected at T1, T3 and T5. Parenting measures were collected and coded at T1, T2, and T3. Temperament was assessed at T2. For an overview of the number of participants with available data on 1, 2 and 3 waves, see Table S1. To test if data were missing at random, we tested for differences on demographical variables (age, sex, psychiatric diagnosis and IQ) between participants with and without data at each time point (see Supplementary Materials).

**Table 1:** Demographic characteristics of the complete samples at T1, T2, T3 and T5.

	**T1**	**T2**	**T3**	**T5**
*N*	512	494	456	336
Age (SD) in years	7.95 (0.67)	8.94 (0.67)	9.98 (0.69)	12.41 (0.76)
Age range	7.02 – 9.68	7.96 – 10.67	8.97 – 11.67	11.15 – 14.11
Girls (%)	51.2	51.0	52.2	52.4
SES* : low-middle high (%)	8.6 – 45.7 – 45.3	8.5 – 45.7 – 45.3	6.6 – 46.1 – 46.9	3.6 – 46.4 – 49.4
Psychiatric diagnosis (n)	11	- * **	16	19
- ADHD/ADD	9	-	9	9
- ADHD/ADD & DCD	0	-	1	1
- DCD	0	-	0	1
- ASD	1	-	3	4
- GAD	1	-	2	2
- OCD	0	-	0	1
- PTSD	0	-	1	0
- Tourette’s syndrome	0	-	0	1
Mean IQ* * (SD)	103.58 (11.76)	103.77 (11.67)	103.81 (11.63)	104.29 (11.89)
IQ range	72.50 – 137.50	72.50 – 137.50	72.50 – 137.50	72.50 – 137.50
Monozygotic (%)	54.7	55.1	54.4	56.0
Caucasian ethnicity (%)	90.2	89.9	89.9	91.1
Age (SD) primary parent	40.95 (4.57)	42.01 (4.62)	43.03 (4.68)	45.85 (4.72)
Age range primary parent	29.75 – 54.83	30.72 – 55.82	31.75 – 56.90	35.24 – 59.21
Female (%) primary parent	91.4	91.1	91.2	90.5

*Note.* * Socio economic status (SES), based on parental education at T1. SES data of 1 family (2 participants) is missing. * * Intelligence quotient, measured at T1 with the subtests “similarities” and “block design” of the WISC (3rd edition). * ** At T2, no information on psychiatric diagnoses was collected. Abbreviations: ADHD/ADD = attention deficit (hyperactivity) disorder, DCD = developmental coordination disorder, ASD = autism spectrum disorder, GAD = generalized anxiety disorder, OCD = obsessive compulsive disorder, PTSD = post-traumatic stress disorder.

### 2.2 Procedure

Data collection took place during annual visits with alternating lab/MRI (T1, T3, T5) and home visits (T2). At T1, T3 and T5, children and their primary parent (i.e., the parent that spent most time with their children at the start of the study) were invited to the Leiden University Medical Center (LUMC). During lab visits, the order of task administration was counterbalanced: one child of the twin pair first participated in an MRI session (including the Social Network Aggression Task), while the other first performed behavioral tasks, filled out questionnaires and participated in observational interaction tasks (including the Etch-a-Sketch) with the primary parent. Thereafter, the children switched and the other child within the twin pair participated in an MRI session, while the other performed behavioral tasks, filled out questionnaires and participated in the observational interaction tasks with the primary parent. Behavioral and MRI data were collected for both children. Both parents filled out questionnaires in Qualtrics prior to or during the visit. Home visits followed a similar set-up, where children also participated in behavioral tasks, parent-interaction tasks and questionnaires.

Between T2 and T3, a Video-feedback Intervention to promote Positive Parenting and Sensitive Discipline (VIPP-SD) took place (Euser et al., 2016, Runze et al., 2022). In between the two sessions, 37% of the sample was randomized to the intervention and 63% to the control group. The intervention consisted of five bi-weekly sessions where parents received video-feedback on interactions with their child. The control condition consisted of five phone calls to the parents. To focus on the development of parenting behavior independent of possible intervention effects, we controlled for the VIPP-SD in our analyses conform our preregistration.

### 2.3 Behavioral measures

#### 2.3.1 Social network aggression task

**Fig. 2:**
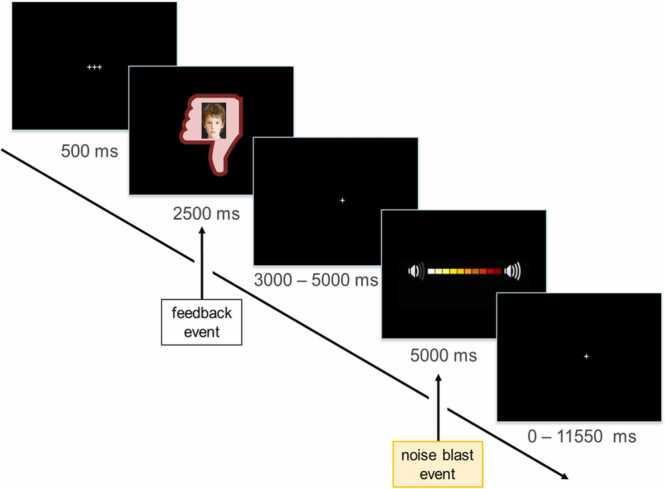
Schematic representation of a trial in the Social Network Aggression Task in the negative social feedback condition. Analyses in this paper are focused on behavior and neural activation at the noise blast event.

The Social Network Aggression Task (SNAT) has previously been validated as reliable measure of behavioral control following social feedback (Achterberg et al., 2016, Achterberg et al., 2017). Participants filled out a personal profile prior to each lab visit, with questions such as ‘what is your favorite sport?’ and ‘what is your favorite subject in school?’, reflecting their personal preferences at that specific time point. During the instruction of the task, participants were told that other peers had evaluated their profile and indicated whether they liked their answers (displayed as green thumb up in the task), disliked their answers (displayed as red thumb down) or did not know whether they liked their answers (displayed as grey round circle). Participants were not made aware of the fact that these peers were not real, but morphed photographs. During the SNAT, participants were first presented with the peer feedback. Subsequently, they were instructed to indicate the duration of a noise blast by pressing a button, imagining it would be sent to the peer. A longer button press was indicative of increased sound and duration of the noise blast, as displayed in a volume bar where a new colored box appeared every 350 ms. A trial consisted of a fixation screen for 500 ms, a social feedback screen for 2500 ms, a jittered fixation screen for 3000–5000 ms, the noise blast screen (with the volume bar) for 5000 ms and an intra-trial fixation screen for 0–11550 ms (Fig. 2). When participants released the button during the noise blast screen, or after 3500 ms, the volume bar was presented for the remaining of the 5000 ms. When participants did not press the button within 1500 ms, a screen with the text ‘too late!’ would appear and these trials were regarded as invalid.

Prior to the MRI session, participants practiced six trials of the task, with each social feedback condition presented twice. During the practice session, participants were presented with the sound of the volume bar twice: once with increasing volume for each colored block, and once with the maximum volume. During the actual task, they did not hear any sound but were instructed to imagine it. The SNAT consisted of sixty trials divided over three blocks of twenty trials. The trials were presented in a pseudo-randomized order, such that no more than three trials with the same feedback (positive, negative, neutral) were presented in a row. For each feedback condition, twenty trials were presented in total.

Behavioral control following social feedback in the SNAT was measured as the difference in noise blast duration following negative feedback and noise blast duration following positive feedback (Dobbelaar, Achterberg, van Drunen et al., 2022). In secondary analyses, we additionally explored the difference in noise blast duration following negative feedback and noise blast duration following neutral feedback.

#### 2.3.2 Etch-a-Sketch

To observe parental sensitivity in a demanding, structured cooperative play situation, we used a computerized version of the Etch-a-Sketch task (Cents et al., 2014). The primary parent performed the task with each of the twin children separately. During the Etch-a-Sketch, the parent and child were instructed to make three drawings on the computer. The specific drawings were printed and presented in order of increasing difficulty. The parent and child both controlled two buttons, such that one could draw vertical lines and the other could draw horizontal lines. To draw diagonal lines, they had to cooperate by pressing the buttons simultaneously. In total, the task lasted eight minutes. Four minutes after the start of the task, an audio message was presented, instructing the participants to continue with the second drawing if they had not yet done so. The parent-child interaction during the task was filmed and the drawings on the computer screen were recorded as well. Both recordings were combined into one video that was coded by trained coders.

Parental sensitivity during the Etch-a-Sketch was coded with scales for supportive presence and intrusiveness (Egeland et al., 1990, Euser et al., 2020). For both scales, a 7-point rating scale was used (supportive presence: 1 =‘Parent completely fails to be supportive to the child’, 7 =‘Parent skillfully provides support throughout the session’; intrusiveness: 1 =‘Parent allows the child sufficient time to explore and attempt to solve tools on his/her own’, 7 =‘Parent is highly intrusive; her/his agenda clearly has precedence over the child wishes’). Videos were coded on supportive presence and intrusiveness by thirteen coders, who were trained by two expert coders. Parenting in different assessment waves were coded by different raters, using the same coding scheme. Intercoder reliability between the expert coder and among coders ranged between ICCs of.71 and.78 across the three time points (Runze et al., 2022). Intrusiveness scores were reversed coded, such that a higher score on both scales represent more parental sensitivity.

The supportive presence and intrusiveness scales were highly correlated (T1: *r* = 0.61, *p* < .001; T2: *r* = 0.53, *p* < .001; T3: *r* = 0.55, *p* < .001) and were therefore combined into one average measure of parental sensitivity.

### 2.4 Neural measures

#### 2.4.1 fMRI acquisition

MRI scans were acquired on the same Philips Ingenia 3.0 Tesla MR scanner for the three waves of fMRI data collection. A standard whole-head coil was used, with foam inserts added to minimize head motion. A screen was placed behind the MRI scanner, such that participants could view the screen displaying the stimuli through a mirror on the head coil. T2 * -weighted echo planar imaging (EPI) was used to collect the fMRI scans. The first two volumes were discarded to allow for equilibration of T1 saturation effects (Field of View (FOV)= 220×220× 111.65 mm; TR= 2.2 s, TE= 30 ms, FA= 80°; sequential acquisition; 37 slices; voxel size= 2.75 × 2.752.72.75 mm). A high-resolution 3D T1 scan was collected as anatomical reference (FOV=224×177×168 mm; TR=9.72 ms; TE=4.95 ms; FA=8°; 140 slices; voxel size=0.875 ×0.875 ×0.875 mm).

#### 2.4.2 fMRI preprocessing

fMRI data were analyzed in SPM12 (Wellcome Department of Cognitive Neurology, London). Images were corrected for slice timing acquisition and rigid body motion. The next step included spatial normalizing to T1 templates (based on MNI-305 stereotaxic space; Cocosco et al., 1997) using 12-parameter affine transform mapping and non-linear transformation with cosine basis functions. Volumes of each participant were resampled to 3×3×3 mm voxels and were spatially smoothed using a 6 mm full-width-at-half-maximum isotropic Gaussian kernel. Data of participants with at least two blocks of fMRI data with < 3 mm movement in every direction on a specific time point were included in the analyses.

#### 2.4.3 fMRI whole-brain analyses

Individual participant’s data at each wave were analyzed using a general linear model in SPM12. Analyses in this paper were focused on activation during the noise blast event. To model the start of noise blast, the hemodynamic response function (HRF) was modeled for the length of the noise blast duration. Noise blasts following positive, neutral and negative feedback were modeled as separate regressors (Achterberg et al., 2018). Trials on which participants did not respond in time were marked invalid and excluded from further analyses. Six motion regressors were added as covariates of no interest. Least-squares parameter estimates (PEs) of height of the best fitting canonical HRF for each condition were used in pairwise contrasts. We used the resulting subject-specific contrast images in group-level analyses. Feedback effects during the noise blast event were investigated using a full-factorial ANOVA with three levels (noise after feedback: “PositiveNoise”, “NeutralNoise”, “NegativeNoise”). We specifically explored the following contrasts in whole-brain analyses: “PositiveNoise>NegativeNoise”, “NegativeNoise>PositiveNoise”, “NeutralNoise>NegativeNoise” and “NegativeNoise>NeutralNoise”. All results were family-wise error (FWE) cluster-level corrected (P_FWEcc_<0.05) with an initial voxel-wise threshold of *p* < .001 (uncorrected). Coordinates for local maxima are reported in MNI space. Untresholded statistical maps of the whole-brain contrasts are available on Neurovault (Gorgolewski et al., 2015) via https://neurovault.org/collections/UKNZFSQB/.

#### 2.4.4 fMRI region of interest analyses

Based on our prior findings that DLPFC activation during noise blast is related to decreased noise blast duration in middle childhood (Dobbelaar, Achterberg, van Drunen et al., 2022), we selected the dorsolateral prefrontal cortex (DLPFC) as region of interest. In our primary analyses, we used the right DLPFC activation during noise blast in T5 as our ROI. We selected T5 since our outcome measure (i.e., responses to social feedback) was measured at this time point. To construct our ROI, clusters of activation from the whole-brain contrast “PositiveNoise > NegativeNoise” were masked with the DLPFC region from the Automated Anatomical Labeling atlas (Tzourio-Mazoyer et al., 2002).

Additionally, in a preregistered secondary analysis and as a robustness check, we tested our models using an independent ROI. Van de Groep et al. (2021) used the SNAT in a sample of young adults and found left DLPFC activation during noise blasts following positive vs. negative feedback and following positive vs. neutral feedback. We used the overlap between the two contrasts in left DLPFC activation from this study as an independent ROI (see Van de Groep et al., 2021). We found similar results as in the analyses using the DLPFC ROI of T5 (see supplementary materials).

Parameter estimates were extracted using the MarsBar toolbox (Brett et al., 2002) for the contrasts “PositiveNoise>NegativeNoise” and “NeutralNoise>NegativeNoise”, which were used as measure of neural activity during responses to social feedback (Dobbelaar, Achterberg, van Drunen et al., 2022). Additionally, parameter estimates were extracted for the separate contrasts “PositiveNoise > fixation”, “NeutralNoise > fixation” and “NegativeNoise > fixation”, to explore developmental effects in activation during behavioral response for each feedback condition.

### 2.5 Stability of responses to social feedback, parental sensitivity and neural measures

To test for the stability of the longitudinal measures (Etch-a-Sketch, SNAT behavioral and DLPFC parameter estimate difference score) over time, we inspected the intraclass correlation coefficients (ICC). Intraclass correlation coefficients (ICC) were calculated using the single measure of the two-way mixed effect model (ICC3,1) consistency definition. For the SNAT and DLPFC parameter estimates we calculated the ICC across T1, T3 and T5. For the Etch-a-sketch we calculated the ICC across T1, T2 and T3. ICC’s above 0.1 were interpreted as notable nesting of observations within individuals (Ordaz et al., 2013).

ICCs for noise blast durations were above 0.1 (negative–positive feedback: ICC = 0.21, 95%CI [0.15,0.27]; negative – neutral feedback: ICC = 0.16, 95%CI [0.11,0.22]; negative feedback: ICC = 0.19, 95%CI [0.14,0.25]; positive feedback: ICC = 0.30, 95%CI [0.25,0.36]). ICCs for parental sensitivity were also above 0.1 and showed fair consistency (Cicchetti, 1994; ICC = 0.44, 95%CI [0.38,0.49]). Finally, the ICC for the difference score in DLPFC activity (positive-negative) was not above 0.1 (ICC = 0.10, 95%CI [0.05,0.16]), but the ICCs for the separate contrasts and the difference score between neutral and negative were (positive feedback: ICC = 0.16, 95%CI [0.10, 0.21]; negative feedback: ICC = 0.11, 95%CI [0.05,0.16]; neutral-negative: ICC=0.11, 95%CI [0.06,0.17]).

### 2.6 Statistical analyses

Outliers were defined as Z-scores below − 3.29 or above 3.29 and those data points were winsorized (Tabachnick et al., 2007), in accordance with our preregistration.

#### 2.6.1 Preregistered analyses

Confirmatory analyses were preregistered on the Open Science Framework (https://osf.io/km32e). Linear mixed models were performed in R (version 4.1.3; R Core Team, 2013). Linear growth curve modeling was performed in Mplus (version 8.7; Muthén and Muthén, 2017). An overview of methodological deviations from the preregistration are presented in the Supplementary Materials. As a sensitivity check, we accounted for non-independence of twins within families by testing our analyses in two randomly split samples, that both included one child per twin pair. Directions and effect sizes of significant effects were comparable to the results using linear mixed models. Results of the split-sample analyses are reported in Table S8, Table S9 and Table S10.

**Development of responses to social feedback.** To examine the development of responses following social feedback (both on a behavioral and neural level), we tested for main effects of time point (T1, T3, T5) and feedback condition (positive, neutral, negative), and feedback condition * time point interaction effects on noise blast duration and DLPFC activation during noise blasts (parameter estimates) using a linear mixed model. We added sex and VIPP-SD as covariates and included two random intercepts to control for nesting of conditions within children and children within families. Thus, our linear mixed model was defined in R as: noise / DLPFC activation ∼ condition*time point + condition*VIPP-SD + condition*sex + (1|ChildID) + (1|FamilyID). We used Type III ANOVAs with Satterthwaite’s method and post-hoc investigated significant effects with Kenward-Roger corrected degrees of freedom and Bonferroni-adjusted *p*-values. As a sensitivity check, we checked whether the addition of zygosity as covariate would affect the results, but all effects remained similar.

**Correlations.** As preregistered, correlations between all variables analyses were calculated using Pearson correlation coefficients.

**Association parental sensitivity and responses to social feedback.** To test whether the development of parental sensitivity was associated with increased inhibitory responses following social feedback in early adolescence, we tested whether the intercept and slope of parental sensitivity were predictive of responses to social feedback at T5. The results of these analyses are reported in the supplementary materials.

Additionally, we tested whether the starting point (intercept at T1) and development (slope) of both parental sensitivity and responses to social feedback were associated using bivariate growth curve models. Both intercepts of parental sensitivity and noise blast duration / DLPFC activation during noise blasts were set at T1. However, because results indicated that the between-person variance of the estimated slope of parental sensitivity was negative, the variance was set to 0, indicating no between-subject differences in linear slope. As such, the slope could not be used as predictor. Thus, within the bivariate growth curve models, we tested for intercept-intercept and intercept-slope relations between parental sensitivity and responses to social feedback (see Fig. S1b). We performed four bivariate growth curve models with noise blast duration (difference score negative-positive, negative feedback, and positive feedback) and DLPFC activation during noise blasts (difference score positive-negative) as outcome variables. In non-preregistered secondary analyses, we additionally explored the bivariate growth curve models with noise blast duration difference score negative-neutral and DLPFC activation difference score neutral-negative. First, we tested the models with covariances between all intercepts and slopes to explore associations. For significant covariances between parental sensitivity and noise blast duration / DLPFC activation, we subsequently tested whether the parental sensitivity variable was predictive of responses to feedback, controlled for VIPP-SD and sex. FamilyID was added as clustering variable to Mplus and missing data were handled using full information maximum likelihood (FIML) with MLR estimators. In addition, we tested the bivariate growth curve models using latent basis growth curve modeling to capture non-linear slopes. Results revealed similar associations between parental sensitivity and responses to social feedback compared to the linear bivariate growth curve models (see supplementary materials).

**Moderation effects of temperament.** We tested whether temperament moderated the relation between parental sensitivity and responses to social feedback. We did not find moderation effects. These analyses are reported and discussed in the supplementary materials.

#### 2.6.2 Exploratory analyses

**Whole-brain analyses.** In addition to our preregistered ROI analyses, we investigated the whole-brain contrasts “PositiveNoise > NegativeNoise”, “NegativeNoise > PositiveNoise”, “NeutralNoise > NegativeNoise” and “NegativeNoise > NeutralNoise” at T1, T3 and T5 to explore activation during noise blasts in neural regions outside the DLPFC.

**Whole-brain regression analyses.** Additionally, we tested whether neural activation during behavioral responses to feedback was related to noise blast durations as an individual differences measure, by performing two exploratory multiple regression analyses: one on the whole-brain contrast “PositiveNoise > NegativeNoise” with the difference in noise blast duration following negative versus positive feedback (Δ negative-positive) as regressor of interest, and one on the whole-brain contrast “NeutralNoise > NegativeNoise” with the difference in noise blast duration following negative versus neutral feedback (Δ negative-neutral) as regressor of interest. Results were FWE cluster-level corrected (P_FWEcc_<0.05) with an initial voxel-wise threshold of *p* < .001.

## 3 Results

### 3.1 Preregistered analyses

#### 3.1.1 Development of responses to social feedback

**Fig. 3:**
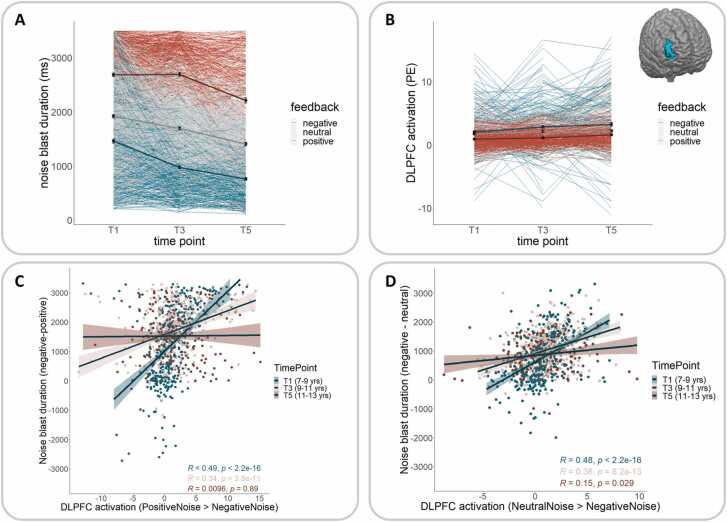
Developmental trajectories across T1, T3 and T5 for A) noise blast duration following positive (in blue), neutral (in grey) and negative (in red) feedback; B) DLPFC activation during responses to positive (in blue), neutral (grey) and negative (in red) feedback. C) Brain-behavior correlations between DLPFC activation (PositiveNoise>NegativeNoise) and noise blast duration (Δ negative–positive) at T1, T3 and T5. D) Brain-behavior correlations between DLPFC activation (NeutralNoise>NegativeNoise) and noise blast duration (Δ negative-neutral) at T1, T3 and T5.

**Noise blast duration.** For noise blast durations, there was a main effect of condition (*F*(2,3335.2)= 1655.49, *p* < .001), showing longest noise blasts following negative feedback, followed by shorter noise blast following neutral feedback and shortest noise blast following positive feedback (all *p* < .001; Fig. 3a). There was also a main effect of time point (*F*(2,3461.4)= 265.76, *p* < .001), indicating longest noise blast durations at T1, followed by shorter noise blast durations at T3 and shortest noise blast durations at T5 (all *p* < .001). Moreover, there was a significant interaction between condition and time point (*F*(4,3335.2)= 19.15, *p* < .001): for positive and neutral feedback, there was a decrease in noise blast duration between T1 and T3 (*p* < .001), that continued between T3 and T5 (*p* < .001). For negative feedback, however, there was no significant change between T1 and T3 (*p* = 1), but a decrease between T3 and T5 (*p* < .001). Thus, developmental patterns differed for responses to negative compared to positive and neutral feedback (Fig. 3a).

Furthermore, when testing for time point effects using the difference score of negative-positive feedback, noise blast difference score duration at T3 was significantly higher than at T1 and at T5. Noise blast difference score duration at T5 was higher than at T1 (all *p* ≤ .001). When testing for time point effects using the difference score of negative-neutral feedback, noise blast difference score duration at T3 was significantly higher than at T1 and at T5 (both *p* ≤ .001), but noise blast duration at T1 and T5 did not differ from each other. These findings indicate that the difference score is affected by the different longitudinal trajectories of responses to positive, neutral and negative feedback. Therefore, in addition to the difference score analyses, in exploratory analyses, we also tested our models on parental sensitivity and moderation effects with noise blast duration following positive and following negative feedback separately.

**DLPFC activation.** For DLPFC activation, there was a main effect of condition (*F*(2,2403.54)= 84.69, *p* < .001), indicating highest DLPFC activation during behavioral responses in the positive feedback condition, followed by lower DLPFC activation in the neutral condition and lowest activation in the negative feedback condition (all *p* < .001). Additionally, there was a main effect of time point (*F*(2,2745.10)= 27.32, *p* < .001): DLPFC activation for all conditions relative to fixation was lowest at T1, followed by higher activation at T3 and highest DLPFC activation at T5 (all *p* ≤ .005). Moreover, there was a significant interaction between feedback condition and time point, *F*(4,2403.54)= 2.68, *p* = .030; Fig. 3b): in the positive and neutral conditions, DLPFC activation increased between T1 and T3 (positive: *p* < .001; neutral: *p* = .041) but did not significantly change between T3 and T5 (both *p* ≥ .058). In the negative condition, DLPFC activation did not significantly change between T1 and T3 (*p* = .566), but increased between T3 and T5 (*p* = .029). Thus, similar to our behavioral findings, we report differential development trajectories for DLPFC activation during behavioral responses to negative compared to positive and neutral feedback.

#### 3.1.2 Correlations

Correlations between the measures are presented in Table S2. Noise blast duration in the SNAT (negative-positive) was correlated with DLPFC activation during noise blasts (PositiveNoise-NegativeNoise) at T1 (*r* = 0.49, *p* < .001) and at T3 (*r* = 0.34, *p* < .001), but there was no significant correlation at T5 (*r* = 0.01, *p* = .887; Fig. 3c).

We performed exploratory correlation analyses to examine the absence of the brain-behavior correlation at T5. This analysis revealed that at T5, DLPFC activation (PositiveNoise-NegativeNoise) negatively correlated with noise blast duration following positive feedback (*r* = −0.17, *p* = .012) and following neutral feedback (*r* = −0.15, *p* = .022). However, noise blast duration following negative feedback was not correlated with DLPFC activation at T5 (*r* = −0.08, *p* = .236). All other correlations are presented in Table S2.

In supplementary analyses, we additionally explored correlations when using the difference score between negative and neutral feedback. Noise blast duration in the SNAT (negative – neutral) was correlated with DLPFC activation during the noise blast (neutral – negative) at T1 (*r* = 0.48, *p* < .001), at T3 (*r* = 0.36, *p* < .001) and at T5 (*r* = 0.15, *p* = .029). The correlation coefficients significantly differed at all three time points, such that correlations became significantly smaller over time (all *p* ≤ .047; Fig. 3d).

#### 3.1.3 Associations parental sensitivity and responses to social feedback

**Fig. 4:**
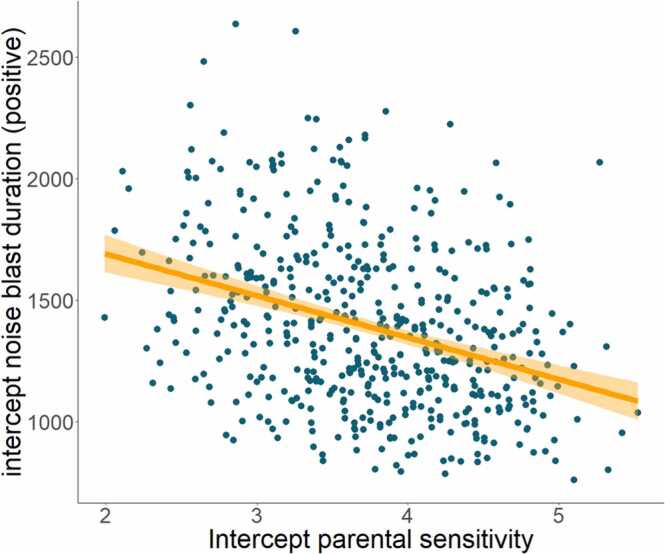
Association between the intercept of parental sensitivity and the intercept of noise blast duration following positive feedback: children of more sensitive parents showed shorter noise blast durations following positive feedback at the first measurement (7–9-years-old).

We did not find predictive effects of parental sensitivity on behavioral responses to social feedback at T5 (see supplementary materials). Results of the bivariate growth curve model revealed that the intercept of parental sensitivity was predictive of the intercept of noise blast duration following positive feedback (β = −0.223, *p* = .011, 95%CI[−0.394, −0.052]; controlled for VIPP and sex): participants with more sensitive parents at T1 showed shorter noise blast durations following positive feedback at T1 (Fig. 4). We did not observe intercept-intercept or intercept-slope relations between parental sensitivity and the other measures of responses to social feedback (see Supplementary Results).

### 3.2 Exploratory analyses

In addition to the preregistered analyses, we performed subsequent non-preregistered exploratory analyses to further examine the development of neural activity to responses to feedback across age.

#### 3.2.1 Whole-brain analyses

**Fig. 5:**
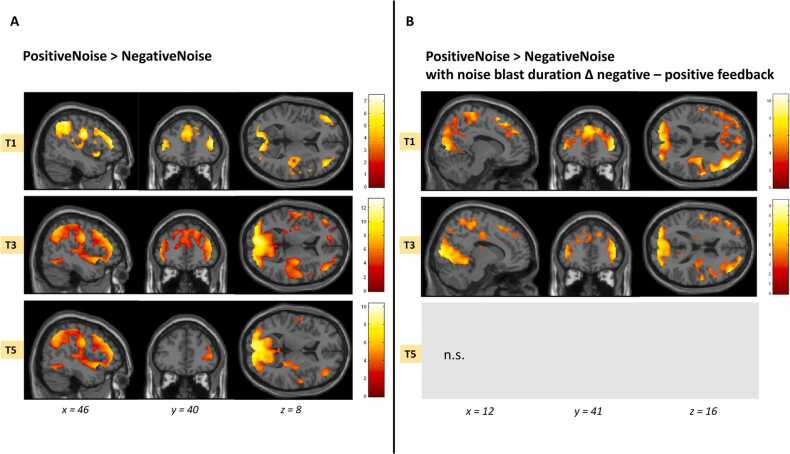
A) Whole-brain activation at T1, T3 and T5 for the contrast PositiveNoise>NegativeNoise. B) Whole-brain regression for the contrast PositiveNoise>NegativeNoise with the difference in noise blast duration between negative and positive feedback as regressor. Results were FWE cluster-level corrected (P_FWEcc_<.05). *n.s.* = not significant.

Whole brain analyses allow us to examine neural activity in regions outside the a priori selected ROIs. We therefore explored feedback effects at each time point for the contrasts “PositiveNoise>NegativeNoise” and “NegativeNoise>PositiveNoise” (Table S7 and Fig. 5a). At T1, the “PositiveNoise>NegativeNoise” contrast resulted in a wide network of activation including the medial and lateral prefrontal regions, insula and occipital gyrus. For the “NegativeNoise>PositiveNoise” contrast, we observed activation in the left postcentral gyrus. At T3, the “PositiveNoise>NegativeNoise” contrast again revealed a wide network of activation including the occipital gyrus and middle temporal gyrus. The “NegativeNoise>PositiveNoise” contrast showed activation in the left postcentral gyrus, supplementary motor area and cingulate cortex. At T5, for the “PositiveNoise>NegativeNoise” contrast we observed a network of activation including the calcarine gyrus, middle frontal gyrus and cerebellum. The reversed contrast did not result in significant clusters of activation.

In supplementary analyses, we explored the contrasts ‘NeutralNoise>NegativeNoise’ and ‘NegativeNoise>NeutralNoise’ at each time point (Table S7). At T1, the ‘NeutralNoise>NegativeNoise’ contrast resulted in a network of activation including the lateral prefrontal cortex, pre- and postcentral gyrus and inferior parietal lobule. The reversed contrast (‘NegativeNoise-NeutralNoise’) did not result in significant clusters of activation. At T3, the ‘NeutralNoise>NegativeNoise’ contrast resulted in activation in the medial and lateral prefrontal cortex, parietal lobule, right cingulate cortex, right SMA and left occipital gyrus. The reversed contrast ‘NegativeNoise>NeutralNoise’ showed activation in the middle temporal gyrus, precuneus and left occipital gyrus. At T5, the ‘NeutralNoise>NegativeNoise’ contrast resulted in activation in the left lingual gyrus and calcarine gyrus. The reversed contrast did not result in significant clusters of activation.

#### 3.2.2 Whole-brain regression

In addition, we performed whole-brain regressions at the three time points on the contrast “PositiveNoise>NegativeNoise” with the difference in noise blast duration following negative and positive feedback as regressor of interest (Δ negative-positive). At T1, we found a positive association between noise blast duration (negative-positive) and neural activation in a network of regions including the dorsolateral prefrontal cortex, inferior frontal gyrus, left insula, parietal regions and the left postcentral gyrus. At T3, we found a positive association between noise blast duration (negative-positive) and neural activation in a similar network of regions. At T5, we did not observe significant clusters of activation for the difference scores (Fig. 5b).

Finally, we performed whole-brain regressions at the three time points on the contrast “NeutralNoise > NegativeNoise” with the difference in noise blast duration following negative and neutral feedback as regressor of interest (Δ negative – neutral). Similar to the results on the PositiveNoise > NegativeNoise contrast, at T1 and at T3, we found a positive association between noise blast duration (negative-neutral) and neural activation in a wide network of regions including the dorsolateral prefrontal cortex, SMA, precentral gyrus and inferior frontal gyrus. At T5, there were no significant clusters of activation. Untresholded statistical maps of the whole-brain contrasts are available on Neurovault (https://neurovault.org/collections/UKNZFSQB/).

## 4 Discussion

This study examined developmental patterns and individual differences in the association between neural activity and the development of responding to social feedback, assessed by noise blasts delivery following peer feedback. We found differential developmental trajectories for noise blast duration following positive and negative feedback. Specifically, noise blasts following positive feedback showed the largest developmental reduction between middle and late childhood (7 – 10 years), whereas noise blasts following negative feedback showed the largest developmental reduction between late childhood and early adolescence (10 – 13 years). The dorsolateral prefrontal cortex, a region previously related to behavioral control following social feedback in childhood (Dobbelaar, Achterberg, van Drunen et al., 2022), showed most activation during noise blasts after positive relative to negative feedback at all time points. This activation was correlated with noise blast differentiation in middle and late childhood, but less strongly in early adolescence. Additionally, a higher level of parental sensitivity predicted shorter noise blasts after positive feedback, validating the relation between behavioral control and observed parental sensitive behavior. However, parental sensitivity did not predict duration of noise blasts or associated neural activity later in time.

### 4.1 Development of behavioral responses to social feedback

This study examined the development of behavioral control following social feedback in an important transition time from childhood to early adolescence. In line with findings on the development of behavioral control in non-social contexts from childhood to adolescence (Crone and Steinbeis, 2017, Luna et al., 2010, Zelazo and Carlson, 2012), we found that noise blast durations decreased with increasing age, possibly indicating an increase in behavioral control in social contexts. Interestingly, this pattern showed a non-linear pattern with highest differentiation between positive and negative feedback in noise blast duration in late childhood (ages 9–11 years), relative to younger (7–9 years) and older (11–13 years) ages. Furthermore, separate analyses revealed a faster decline in noise blast development for positive and neutral compared to negative peer feedback from childhood to adolescence. Several explanations may account for these differential developmental processes. First, decreases in noise blast duration following positive and neutral feedback may reflect development in inhibition processes, that increase between middle and late childhood (Crone and Steinbeis, 2017, Huizinga et al., 2006). Second, middle and late childhood are marked by the internalization of fairness norms and learning to act upon those norms (House, 2018, McAuliffe et al., 2017, Smith et al., 2013) and increases in reciprocity (van den Bos et al., 2010). Children may therefore differentiate more in their responses to positive and negative feedback around ages 9–11-years, whereas young adolescents may be more lenient when it comes to fairness principles associated with an increase in perspective taking (Crone, 2013, Rodman et al., 2017). Third, our finding that noise blasts following negative feedback showed largest reductions between late childhood and early adolescence fits with prior work showing a decrease in reactive aggression from late childhood onwards (Cui et al., 2016, Fite et al., 2008). Being able to resist aggressive responses following negative feedback might aid in achieving social inclusion in the peer group. This need for social inclusion is especially salient in early adolescence given the increased sensitivity to social evaluation and belonging (Blakemore and Mills, 2014, Somerville, 2013). Indeed, previously, adolescents were found to increasingly internalize peer rejection and refrain from self-protective processes (Rodman et al., 2017). Thus, inhibitory control development, fairness principles and an increased need for social belonging in adolescence could possibly be important factors in the development of responding to feedback from childhood towards adolescence.

### 4.2 Neural mechanisms of responding to social feedback

On a neural level, in line with prior work in children and adults (Dobbelaar et al., 2022, van de Groep et al., 2021, van de Groep et al., 2022), activation in the DLPFC was higher during behavioral responses to positive compared to negative feedback, possibly indicating inhibition processes, given that the noise blasts were mandatory and participants aimed for the shortest duration after positive feedback (i.e., signaling peer acceptance). The DLPFC has previously been described as an important region for the development of behavioral and cognitive control (Achterberg et al., 2020, Bunge and Zelazo, 2006, Luna et al., 2010). The results of this study support the hypothesis that DLPFC plays an important role in controlling reactive aggression in two ways. First, we observed across ages increased DLPFC activation when delivering noise blasts following positive and neutral versus negative feedback, suggesting more control was required when participants refrained from aggression. Second, brain-behavior correlations revealed that higher DLPFC activation during noise blasts following positive (versus negative) feedback was related to shorter noise blasts following positive (versus negative) feedback in middle and late childhood, suggesting an important role of the DLPFC in inhibition of responses following social feedback. In terms of developmental transitions, we observed significant associations between noise blast duration and DLPFC activation following positive versus negative, and following neutral versus negative feedback at two time points (middle and late childhood), that became significantly smaller towards early adolescence (van de Groep et al., 2021, van de Groep et al., 2022). Consistent with this finding, the whole-brain regressions revealed a larger network of regions, including DLPFC, related to noise blasts following positive (compared to negative) feedback in middle and late childhood, but not in adolescence.

Together, these findings suggest that the role of the DLPFC in inhibitory behaviors following social feedback may change in the transition from childhood to adolescence. This is the first study including a detailed analyses of transition in this age range using large sample sizes. The results fit with prior studies showing a decrease in the recruitment of the DLPFC during inhibitory tasks across development (Booth et al., 2003, Ordaz et al., 2013, Tamm et al., 2002), often accompanied by adult levels of inhibition in adolescence. However, there are also alternative explanations that should be considered. First, the DLPFC has also been implicated in responding to conflicting information (Kim et al., 2010, Zaki et al., 2010), therefore an increase in DLPFC activation could also be indicative of increased conflict in younger children when having to send a noise blast following positive feedback. Second, DLPFC activation has also previously been linked to overall effort (Braver et al., 1997, Ordaz et al., 2013). Exerting inhibitory control may possibly cost more effort in childhood than in adolescence. Third, the wide network of activation in the whole-brain regressions in childhood additionally supports the hypothesis that children may use more diverse strategies when responding to feedback, especially in salient social contexts (Crone and Steinbeis, 2017). Possibly, towards adolescence, inhibition following social feedback may become more goal-oriented (Crone and Dahl, 2012) and rely on different neural processes. Future studies should examine in more detail the separate components of social feedback processing and responses, differentiating positive and negative feedback as separate processes rather than treating them as polar opposites, and follow participants during adolescence into adulthood.

### 4.3 Parental sensitivity and responses to social feedback

The second aim of this study was to examine environmental influences that might affect the development of responding to social feedback. Indeed, parental sensitivity and behavioral responses following positive feedback were cross-sectionally associated in middle childhood (the first time point), such that children of sensitive parents sent shorter noise blasts following positive feedback. Given that behavioral control tendencies were mainly reflected in the positive and not in negative feedback conditions, our results are in line with studies reporting positive associations between sensitive parenting and self-control in middle childhood (e.g., Colman et al., 2006; Gülseven et al., 2021; Sosic-Vasic et al., 2017). Possibly, sensitive parents model constructive behaviors, such as reciprocity, to their children. In turn, children of sensitive parents might be more able and motivated to internalize this modeled behavior (Eisenberg et al., 2005). It is important to note that relations between parenting and child behaviors are often bi-directional (Farley and Kim-Spoon, 2014, Newton et al., 2014). Since we only found a cross-sectional relation between parental sensitivity and noise blast durations following positive feedback in middle childhood, future studies may further focus on the directionality of this effect using within-person designs starting earlier in childhood. Additionally, further investigation of neural activation linking parenting and behavioral control may advance our understanding of mechanisms underlying environmental effects (Kerr et al., 2019).

Contrary to our predictions, parental sensitivity in childhood was not predictive of children’s responses to feedback in adolescence. Towards adolescence, other social environmental influences on child behavior might become of increasing importance in directing behavior. For example, early adolescence is known as period during which adolescents spend more time with peers, more often confirm to peer norms and peer influence increases (Blakemore and Mills, 2014, Brown and Larson, 2009, Molleman et al., 2022, Nickerson and Nagle, 2005, Pinho et al., 2021). Additionally, there may be other parental influences that become important for self-regulation in adolescence, such as parental monitoring (Li et al., 2022). An interesting future direction would be to investigate the relative and possibly changing roles of parents and peers during the transition towards adolescence, especially in the context of responding to peer feedback.

### 4.4 Strengths, limitations and future directions

This preregistered study has several strengths, including the use of a multi-method approach of observational, experimental, neuroimaging and questionnaire data. Furthermore, we used longitudinal measurements in a large sample to study the development of responses to social feedback from childhood to adolescence. This transition period has received relatively little attention in the literature, especially in terms of neural development of social processes. Our study revealed different developmental trajectories for behavioral control following positive and negative feedback and emphasizes the role of parents in this developmental period. However, some limitations should be acknowledged as well. First, because this study included within-person data on three time points we estimated linear trajectories of development in our secondary bivariate growth curve models. It should be noted that our data revealed developmental patterns indicative of non-linear trajectories. We tested both linear and non-linear models and the results revealed similar associations in both models. An important direction is to include more than three time points in future studies, which allows for better capturing these potentially curvilinear longitudinal trajectories. Second, we accounted statistically for nesting of twins within families, but this does not preclude the possibility that there are unique aspects of social development and parent-child interactions in twin families compared to non-twin families that could hinder the generalizability of our results to a broader population (Thorpe and Danby, 2006). Finally, as participants were instructed to send a noise blast after each feedback presentation, an important question for future research is to fully capture the underlying intentions of self-control following positive feedback. The current version of the SNAT paradigm does not differentiate between children who only refrain from aggression and children who additionally show prosocial behavior by intentionally inhibiting noise blasts. In future research, adding a prosocial response option to the task (such as sending a nice song or triumphant sounds) might help in further disentangling these motives.

## 5 Conclusion

In conclusion, this study examined the development of delivering noise blasts in a social context and tested for parental influences on this development. Our findings demonstrate distinctive trajectories in the development of noise blast delivery following positive and negative feedback, where the regulation of responses following positive feedback might be largely in place during late childhood, whereas the regulation of behavioral responses following negative feedback shows developmental changes towards early adolescence. Moreover, our results point towards the DLPFC as important mechanism for inhibitory responses following feedback in childhood. This study revealed that the function of the DLPFC in behaviorally responding to feedback changes throughout development towards adolescence, possibly reflecting more strategic motives. Finally, we found associations between parental sensitivity and inhibitory responses to positive feedback in middle childhood. Together, our findings contribute to our understanding of individual differences in the development of responding to social feedback, and the role of parenting in supporting children’s adaptive coping with social feedback.

## Funding

This work was supported by the Gravitation program of the Dutch Ministry of Education, Culture, and Science and the Netherlands Organization for Scientific Research (NWO grant number 024.001.003).

## CRediT authorship contribution statement

**Simone Dobbelaar:** Conceptualization, Data curation, Formal analysis, Investigation, Project administration, Visualization, Writing – original draft, Writing – review & editing. **Michelle Achterberg:** Conceptualization, Data curation, Investigation, Resources, Supervision, Writing – review & editing. **Anna C.K. van Duijvenvoorde:** Conceptualization, Supervision, Writing – review & editing. **Marinus H. van IJzendoorn:** Conceptualization, Funding acquisition, Methodology, Writing – review & editing. **Eveline A. Crone:** Conceptualization, Funding acquisition, Methodology, Resources, Supervision, Writing – original draft, Writing – review & editing.

## Declaration of Competing Interest

The authors declare that they have no known competing financial interests or personal relationships that could have appeared to influence the work reported in this paper.

## Data Availability

Data, study material and analysis code are available on DataverseNL through https://doi.org/10.34894/JPJV7X. Group-level MRI data are available on Neurovault through https://neurovault.org/collections/UKNZFSQB/.
