# Supplementary material for: Developmental patterns and individual differences in responding to social feedback: A longitudinal fMRI study from childhood to adolescence
Source: Dev Cogn Neurosci. 2023 Jun 10;62:101264. doi: 10.1016/j.dcn.2023.101264 (PMC10285498; doi:10.1016/j.dcn.2023.101264)
Supplement: Supplementary file 1 — Supplementary material [file mmc1.docx]

**Supplementary Methods**

**Participants**

To test if data were missing at random, we tested for differences on demographical variables (age, sex, psychiatric diagnosis and IQ) between participants with and without data at each time point. Included participants at T2 and T3 were younger (at T1) compared to excluded participants at T2 and T3 (*p* ≤ .002). There were no other differences between included and excluded participants. Additionally, we tested for differences between participants with both behavioral and neural SNAT data and participants with behavioral SNAT data only. There were age differences between both groups, such that at T1 and T3, participants included in the MRI analyses were older than participants with behavioral data only, but at T5 participants included in the MRI analyses were younger than participants with behavioral data only (all *p* ≤ .005). Possibly, at T1 and T3 there was more missing data in younger children due to anxiety and movement in the scanner, whereas at T5 there was more missing data in older children due to braces. Also, the MRI sample at T1 included more girls than the sample with behavioral data only (*p* = .022). On the other demographical variables, there were no differences between the behavioral and MRI sample.

**Behavioral measures**

***Temperament***

To study which children might be most susceptible to parental influences, we tested whether temperament moderated the association between parental sensitivity and responses to social feedback. Children who score higher on the temperamental trait perceptual sensitivity are better able to detect slight, low-intensity stimuli in the environment (Rothbart et al., 2001; Slagt et al., 2018), and may therefore be more susceptible to environmental influences (Ellis et al., 2011; Weeland et al., 2017). Therefore, we explored whether the association between parental sensitivity and inhibitory responses would be stronger for children with more perceptual sensitivity compared to their peers (Ellis et al., 2011; Weeland et al., 2017). In a secondary analysis, we also tested for moderation effects of difficult temperament, a more global measure of temperament, that was previously found to explain susceptibility to the environment for better and for worse (Slagt et al., 2016).

Temperamental traits were measured using the three subscales Discomfort, Fear and Perceptual Sensitivity of the Temperament in Middle Childhood Questionnaire (TMCQ; Simonds et al., 2007). The child and both parents completed the TMCQ in Qualtrics during the home visit at T2. The Discomfort subscale consisted of ten items (e.g., ‘Is likely to cry when even a little bit hurt’), the Fear subscale consisted of eight items (e.g., ‘Is afraid of the dark’) and the Perceptual Sensitivity subscale consisted of nine items (e.g., ‘Notices things others don’t notice’). Items were answered on a on a five-point Likert scale, ranging from 1 (‘completely untrue’) to 5 (‘completely true’).

An average score was computed of the nine items of the Perceptual Sensitivity subscale. In our primary, preregistered, analyses, we used this score as temperamental marker of differential susceptibility (Slagt et al., 2018; Weeland et al., 2017). In our secondary analyses, we used the average of the Fear and Discomfort subscales as measure of difficult temperament (Slagt et al., 2016).

Higher scores indicated more perceptual sensitivity and a more difficult temperament. We preregistered to use the child-report subscale when Cronbach’s *α*≥.70, which we defined as sufficient reliability. Because the child-reported perceptual sensitivity scale did not meet this criterium (Cronbach’s *α*=.66), we used parent-reported scores instead (Cronbach’s *α*=.80). For fear and discomfort, we used the child-reported scale (child report: Cronbach’s *α*=.76; parent report: Cronbach’s *α=*.88).

**Preregistered analyses**

In our preregistration, we specified primary analyses on the association with parental sensitivity and moderating effects of perceptual sensitivity, and secondary analyses on the moderating effects of difficult temperament (Dobbelaar et al., 2022).

***Primary analyses***

**Association parental sensitivity and responses to social feedback.** To test whether the development of parental sensitivity was associated with increased inhibitory responses following social feedback in early adolescence, we estimated the intercept (at T1) and slope of parental sensitivity (across T1, T2 and T3) and tested in Mplus whether those were predictive of responses to social feedback at T5 (Figure S1a). However, there were no between-subject differences in linear slope, so we only used the intercept as predictor variable. We performed two separate analyses, with noise blast duration (Δ negative–positive feedback) and DLFPC activation (Δ PositiveNoise-NegativeNoise) at T5 as outcome variables, respectively.

To control for intervention effects, intervention (VIPP-SD; 0=control, 1=intervention) was added to the models as covariate. Furthermore, we added sex of the child as covariate to control for sex effects. We added FamilyID as a clustering variable in Mplus (using the CLUSTER option), to account for nesting of twins within families and we handled missing data using full information maximum likelihood (FIML) with MLR estimators. We report the standardized results from the STDY output, which standardizes all outcome variables and continuous latent variables, but not binary covariates (Mplus User’s Guide).

Model fit was assessed using χ^2^ (*p*>.05), CFI (>.90) and RMSEA (<.08) criteria. Because these criteria were not available in Mplus for models with clustering variables, we report the model fit values for the linear growth curve models without clustering of FamilyID (see Table S3).

Additionally, we tested whether the starting point (intercept at T1) and development (slope) of both parental sensitivity and responses to social feedback were associated using bivariate growth curve models. Both intercepts of parental sensitivity and noise blast duration / DLPFC activation were set at T1. Details regarding the analyses are presented in the main text.

**Moderation effects of perceptual sensitivity.** To test for moderation effects of perceptual sensitivity on the association between parenting and responses to social feedback, temperament (perceptual sensitivity) was added as moderator to the models of parental sensitivity and responses to social feedback in Mplus. We first centered the temperament variable and subsequently created an interaction variable between the intercept of parental sensitivity and temperament. Because the intercept of parental sensitivity was a latent variable, we did not center this variable. Next, we tested whether this interaction term was predictive of noise blast durations and DLPFC activation at T5, controlled for the main effects of intercept and temperament, sex and VIPP.

Given that we tested four primary models (two for the relation between parental sensitivity and responses to feedback and two for the primary moderation analyses), we corrected our results for multiple testing using the Bonferroni procedure for correlated variables ([https://www.quantitativeskills.com/sisa/calculations/bonfer.htm](https://www.quantitativeskills.com/sisa/calculations/bonfer.html)), which takes into account the correlation between outcome variables. The average correlation between noise blast duration (negative-positive) and DLPFC activation (PositiveNoise-NegativeNoise) at T5 was *r*=0.01, yielding a significance level of α=.013.

Finally, for bivariate growth curve models with significant associations between parental sensitivity and responses to feedback, we tested whether temperament moderated this association, by adding the main and interaction effects of parental sensitivity and temperament as predictors to the regression.

***Secondary analyses***

**Moderation effects of difficult temperament.** In preregistered secondary analyses, we repeated the moderation analyses with difficult temperament (i.e., the fear and discomfort subscales) as moderator.

**Independent ROI of DLPFC.** In a preregistered secondary analysis and as a robustness check, we tested our models using an independent ROI. Van de Groep et al. (2021) used the SNAT in a sample of young adults and found left DLPFC activation during noise blasts following positive vs. negative feedback and following positive vs. neutral feedback. We used the overlap between the two contrasts in left DLPFC activation from this study as an independent ROI (see Van de Groep et al., 2021).

**Exploratory analyses**

***Analyses on the difference score of responses following negative and neutral feedback***

In non-preregistered supplementary analyses, we repeated the analyses on associations between parental sensitivity and responding to social feedback, and moderation effects by temperament on noise the difference in responses to negative and neutral feedback (i.e., noise blast duration: negative – neutral; DLPFC activation: NeutralNoise – NegativeNoise).

***Analyses on noise blast durations following positive and negative feedback separately***

In non-preregistered exploratory analyses, we repeated the analyses on associations between parental sensitivity and responding to social feedback, and moderation effects by temperament on the noise blast duration in the positive and negative feedback conditions separately.

***Bivariate growth curve models using latent basis growth curve modeling***

In non-preregistered exploratory analyses, we tested the bivariate growth curve models using latent basis growth curve modeling to capture non-linear slopes. We tested whether a latent basis growth model would capture the developmental trajectories better than the linear growth models, and if so, whether the use of latent basis growth models would alter the results of the bivariate growth curve models. Because we used MLR estimators in our model, we compared the model fit using loglikelihood tests with MLR scaling correction factors.

**Deviations from preregistration**

***Association parental sensitivity and responses to social feedback***

We preregistered to test whether the intercept (at T1) and slope (across T1, T2 and T3) of parental sensitivity were predictive of the intercept (at T5) and slope of noise blast durations / DLPFC activation (across T1, T3 and T5). However, the intercepts did not match the time frame in real life (i.e., in this model it would be assumed that T1 of parental sensitivity co-occurs with T5 of the SNAT) and therefore we chose an alternative test. We now tested whether the intercept and slope of parental sensitivity were predictive of responses to social feedback at T5. Furthermore, to still test for intercept-intercept, intercept-slope and slope-slope relations, in additional analyses we used bivariate growth curve models where both intercepts of parental sensitivity and responses to social feedback were set at T1.

***Slope parental sensitivity***

We preregistered to test whether the intercept and slope of parental sensitivity were predictive of responses to social feedback. Results indicated that there was a significant between-person variance for the intercept of parental sensitivity (*p* < .001), but the between-person variance of the estimated slope of parental sensitivity was negative and was therefore set to 0, indicating no between-subject differences in linear slope. As such, the slope could not be used as predictor. Thus, in our final models we only used the intercept of parental sensitivity as predictor variable (Figure S1a).

***DLPFC ROI***

We preregistered to use the bilateral DLPFC in the “PositiveNoise > NegativeNoise” contrast from Van de Groep et al (2021) as independent ROI in secondary analyses. However, the “PositiveNoise > NegativeNoise” and “PositiveNoise > NeutralNoise” both resulted in activation in the left DLPFC (van de Groep et al., 2021). The overlap in DLPFC activation between the two contrasts was previously used in brain-behavior correlations (van de Groep et al., 2021). Therefore, we decided to also use this overlap between the two contrasts in the left DLPFC only as independent ROI in our secondary analyses. Additionally, because we did not find bilateral DLPFC activation, we could not explore differences between left and right hemisphere in follow-up analyses.

***Model fit***

Model fit was assessed using χ^2^ (*p* > .05), CFI (>.90) and RMSEA (<.08) criteria. Because these criteria were not available in Mplus for models with clustering variables, we report the model fit values for the linear growth curve models without clustering of FamilyID (see Table S3).

Second, the model fit criteria as specified in the preregistration were also not available for models with interaction terms. Therefore, in the moderation analyses, we first checked the model fit for models without interaction term (i.e., with only main effects of parental sensitivity and temperament, see Table S4). Because these models with the sex and VIPP-SD covariates showed poor model fit and the covariates did not significantly affect the outcome measures, we used interaction models without covariates to test for moderation effects (Table S4). We used log-likelihood testing with MLR estimators to check whether the addition of the interaction term improved the model fit compared to models without interaction (Maslowsky et al., 2014). For all moderation models, model fit was significantly improved when adding the interaction term (see Table S5).

**Supplementary Results**

**Preregistered primary analyses**

***Association parental sensitivity and responses to social feedback***

To test whether the development of parental sensitivity was associated with inhibitory responses to social feedback in early adolescence, we tested whether the intercept (at T1) of parental sensitivity was predictive of noise blast duration and DLPFC activation at T5. Notably, the mean of the slope of parental sensitivity was not significantly different from zero (*b*=0.065, *p*=.064), indicating no mean linear development over time points T1-T2-T3 (Figure S2).

**Noise blast duration.** We first tested whether parental sensitivity predicted noise blast duration later in time. The intercept of parental sensitivity at T1 was, however, not associated with noise blast duration (negative–positive) at T5 (β=0.014, *p*=.852; controlled for sex and VIPP).

**DLPFC activation.** Next, we tested whether parental sensitivity predicted neural activity associated with noise blast duration later in time. The intercept of parental sensitivity at T1, however, did not predict DLPFC activation (PositiveNoise–NegativeNoise) at T5 (β=-0.030, *p*=.726; controlled for sex and VIPP).

***Association parental sensitivity and responses to social feedback – bivariate growth curve models***

Second, we tested for intercept-intercept, intercept-slope associations between parental sensitivity and responses to social feedback in bivariate growth curve models. Because there was no between-person variance in the slope of parental sensitivity, we could only explore three associations within the bivariate growth curve model: 1) the association between intercept of parental sensitivity (at T1) and intercept of responses to social feedback (at T1); 2) the association between the intercept of parental sensitivity (at T1) and the slope of responses to social feedback (across T1, T3, T5); and 3) the association between the intercept of responses to social feedback (at T1) and the slope of responses to social feedback (across T1, T3, T5; see Figure S1b).

**Noise blast duration.** There were no significant covariances between parental sensitivity and the difference score in noise blast duration (negative – positive; all *p* > .109).

**DLPFC activation****.** The estimated between-person variances of both the intercept and slope of DLPFC activation were negative and therefore could not be used to calculate covariances in the model. As such, we could not explore associations between the intercepts and slopes of parental sensitivity and DLPFC activation (PositiveNoise – NegativeNoise).

***Moderation effects of perceptual sensitivity***

To test whether the temperamental trait perceptual sensitivity worked as a moderator on the association between the intercept of parental sensitivity and responses to social feedback, we tested whether this interaction term was predictive of noise blast duration and DLPFC activation at T5.

**Noise blast duration.** There was no interaction effect of perceptual sensitivity and the intercept of parental sensitivity (β = -0.018, *p* = .863; see Table S6). Thus, we did not find moderating effects of perceptual sensitivity on the relation between parental sensitivity in childhood and noise blast duration in early adolescence.

**DLPFC activation.** DLPFC activation (PositiveNoise - NegativeNoise) at T5 was not predicted by the interaction of perceptual sensitivity and the intercept of parental sensitivity (β = 0.009, *p* = .919; see Table S6). Thus, there were also no moderating effects of perceptual sensitivity on the relation between parental sensitivity in childhood and DLPFC activation in early adolescence.

**Preregistered secondary analyses**

***Moderation effects of difficult temperament***

We additionally tested whether difficult temperament (i.e., the fear and discomfort subscales) worked as a moderator on the association between the intercept of parental sensitivity and responses to social feedback.

**Noise blast duration.** There was no interaction effect of fear and discomfort and the intercept of parental sensitivity (β = 0.004, *p* = .946; see Table S6). Thus, we did not find moderating effects of difficult temperament on the relation between parental sensitivity in childhood and noise blast duration in early adolescence.

**DLPFC activation.** DLPFC activation (PositiveNoise - NegativeNoise) at T5 was not predicted by the interaction of fear and discomfort and the intercept of parental sensitivity (β = -0.028, *p* = .642; see Table S6). Thus, there were also no moderating effects of difficult temperament on the relation between parental sensitivity in childhood and DLPFC activation in early adolescence.

***Independent ROI of DLPFC (Van de Groep et al., 2021)***

**Correlations.** DLPFC activation during responses to positive – negative feedback (PositiveNoise – NegativeNoise) was significantly correlated to noise blast duration following negative – positive feedback at T1 and T3 (T1: r = 0.32, p < .001; T3: r = 0.23, p < .001), but not at T5 (r = 0.02, p = .805).

**Development DLPFC activation.** For DLPFC activation, there was a main effect of condition (F(1,1499.08) = 66.30, p < .001), indicating higher DLPFC activation during responses to positive compared to negative feedback. Additionally, there was a main effect of time point (F(2,1778.51) = 3.65, p = .026): DLPFC activation was significantly lower at T1 than at T5 (p = .039). There was no significant interaction between feedback condition and time point, F(2,1499.08) = 1.63, p = .196).

**Associations parental sensitivity and responses to social feedback.** We tested whether the intercept of parental sensitivity was predictive of DLPFC activation (positive – negative) at T5. Model fit was sufficient (χ^2^(11) = 12.90, p = .300; CFI = 0.991; TLI = 0.989; RMSEA = 0.019 (95%CI [0.000; 0.053]). The intercept of parental sensitivity at T1 did not predict DLPFC activation at T5 (β = 0.015, p = .861; controlled for sex and VIPP). Additionally, the bivariate growth curve model for DLPFC activation during responses to feedback (PositiveNoise - NegativeNoise) did not reveal significant covariances between parental sensitivity and DLPFC activation (all p > .371).

**Moderation effects of temperament.** DLPFC activation (PositiveNoise - NegativeNoise) at T5 was not predicted by the interaction of temperament and the intercept of parental sensitivity (β = 0.013, p = .863), nor by the interaction of fear and discomfort and the intercept of parental sensitivity (β = 0.048, p = .587).

**Exploratory analyses**

***Analyses on the difference score of responses following negative and neutral feedback***

In non-preregistered supplementary analyses, we repeated our analyses on the difference score of responses following negative and neutral feedback (noise blast duration negative – neutral, DLPFC activation NeutralNoise – NegativeNoise).

**Associations parental sensitivity.** The intercept of parental sensitivity was not predictive of noise blast duration (negative – neutral) and DLPFC activation (NeutralNoise – NegativeNoise) at T5 (Table S3).

**Associations parental sensitivity – bivariate growth models.** For the bivariate growth model on noise blast duration (negative – neutral), there were no significant covariances (all *p* > .306). For the bivariate growth model on DLPFC activation, the estimated between-person variances of both the intercept and slope of DLPFC activation were negative and therefore could not be used to calculate covariances in the model. As such, we could not explore associations between the intercepts and slopes of parental sensitivity and DLPFC activation (NeutralNoise – NegativeNoise).

**Moderation effects of temperament.** We did not find moderation effects of perceptual sensitivity or of fear and discomfort, on the association between the intercept of parental sensitivity and noise blast duration, nor on the association between the intercept of parental sensitivity and DLPFC activation (Table S6).

***Analyses on noise blast durations following positive and negative feedback separately***

In exploratory analyses, we repeated our analyses on parenting associations and moderation effects for noise blast durations in the positive and negative feedback conditions separately.

**Associations parental sensitivity.** Repeating the analyses of whether parental sensitivity predicted noise blast durations at T5 for positive and negative feedback conditions separately did not change the results (Table S3).

**Associations parental sensitivity – bivariate growth curve models.** For the negative feedback condition, there were no significant covariances between parental sensitivity and noise blast duration following negative feedback (all *p* > .576). For the positive feedback condition, the intercept of parental sensitivity was predictive of the intercept of noise blast duration (β = -0.223, *p* = .011, 95%CI [-0.394, -0.052]; controlled for VIPP and sex): participants with more sensitive parents at T1 showed shorter noise blast durations following positive feedback at T1 (Figure 4).

**Moderation effects of temperament.** Repeating the moderation analyses for positive and negative feedback conditions separately also did not alter the results. Both perceptual sensitivity and difficult temperament did not moderate the association between parental sensitivity and noise blast duration following positive and negative feedback (Table S6). Additionally, the intercept-intercept relation between parental sensitivity and noise blast duration following positive feedback (Figure 4) was not moderated by perceptual sensitivity, nor by difficult temperament (Table S6).

***Bivariate growth curve models using latent basis growth curve modeling***

In exploratory analyses, we tested whether latent basis growth curve models would capture the developmental trajectories better than the linear growth curve models. Model fit was improved when using latent basis growth modeling for the parental sensitivity and all behavioral SNAT variables (all *p* ≤ .003), but not for the neural DLPFC variables (both *p* ≥ .123). In the latent basis growth model for parental sensitivity, the variance in slope was still very close to zero (variance estimate = 0.003, *p* = .959).

Subsequently, we tested whether the use of latent basis growth factors (for parental sensitivity and behavioral SNAT variables) would alter the results of the bivariate growth curve models with linear growth factors. Similar to the linear bivariate growth curve models, the only significant association between parental sensitivity and responses to feedback was found for the SNAT positive condition: the intercept of parental sensitivity was positively associated with the intercept of noise blast duration following positive feedback (β = -0.205, *p* = .007, 95CI [-0.354, -0.057]; corrected for VIPP and sex). This association was not moderated by perceptual sensitivity (β = 0.009, *p* = .873), nor by fear and discomfort (β = 0.111, *p* = .078). Although it was not the focus of the bivariate models (where we aimed to test relations between parental sensitivity and responses to feedback), we also found a significant negative association between the intercept and slope of the SNAT in the negative-positive, negative-neutral and positive condition (negative-positive: β = -0.866, *p* < .001; negative-neutral: β = -0.871, *p* < .001; positive: β = -0.926, *p* < .001). We did not find any other significant associations.

**Supplementary Discussion**

**Moderation by temperament**

Individual differences in the association between parental sensitivity and responding to social feedback were not explained by temperament, in contrast to prior findings on externalizing and prosocial behaviors (Slagt et al., 2016, 2018; Stright et al., 2008). Differential susceptibility effects, where a subset of children is more vulnerable in negative environments but thrive in positive environments (Belsky et al., 2007; Ellis et al., 2011), are often reported in early childhood. Both early childhood and adolescence have been suggested as important periods of neurodevelopmental plasticity and increased environmental sensitivity (Guyer et al., 2018), during which some children might be more affected by their social environment than others. Also, negative emotionality, which might be reflected in our fear and discomfort scale, was previously found to explain differential susceptibility, but only when it was assessed during early childhood (Slagt et al., 2016). Our results did not find evidence for the hypothesis that behavioral control development in middle childhood is affected by individual differences in perceptual sensitivity or difficult temperament. Possibly, other measures might better explain individual differences in the relation between parenting and responses to social feedback in middle childhood. For instance, some children may be more susceptible to parenting behavior because of neurobiological susceptibility (Schriber & Guyer, 2016) or genetic make-up (Belsky & van IJzendoorn, 2017). Additionally, differential susceptibility might best be tested in changing environments, that is, when parental sensitivity increases or decreases over time. Because we did not find between-person variation in the linear slope of parental sensitivity, we could not test for associations with *change* in parental sensitivity. However, between-person differences in the intercept of parental sensitivity might indirectly still be indicative of developmental processes in early childhood. Future research on other potential markers might shed some light on individual differences in environmental susceptibility effects during middle childhood.

**Supplementary References**

Belsky, J., Bakermans-Kranenburg, M. J., & van IJzendoorn, M. H. (2007). For Better and For Worse: Differential Susceptibility to Environmental Influences. *Current Directions in Psychological Science*, *16*(6), 300–304. https://doi.org/10.1111/j.1467-8721.2007.00525.x

Belsky, J., & van IJzendoorn, M. H. (2017). Genetic differential susceptibility to the effects of parenting. *Current Opinion in Psychology*, *15*, 125–130. https://doi.org/https://doi.org/10.1016/j.copsyc.2017.02.021

Dobbelaar, S., Achterberg, M., van Duijvenvoorde, A. C. K., van IJzendoorn, M. H., & Crone, E. A. (2022). Differential susceptibility of associations between parental sensitivity and social behavioral control: a longitudinal fMRI design. In *Open Science Framework*. https://doi.org/https://doi.org/10.17605/OSF.IO/SC4K8

Ellis, B. J., Boyce, W. T., Belsky, J., Bakermans-Kranenburg, M. J., & van Ijzendoorn, M. H. (2011). Differential susceptibility to the environment: An evolutionary–neurodevelopmental theory. *Development and Psychopathology*, *23*(1), 7–28. https://doi.org/DOI: 10.1017/S0954579410000611

Guyer, A. E., Pérez-Edgar, K., & Crone, E. A. (2018). Opportunities for Neurodevelopmental Plasticity From Infancy Through Early Adulthood. *Child Development*, *89*(3), 687–697. https://doi.org/https://doi.org/10.1111/cdev.13073

Maslowsky, J., Jager, J., & Hemken, D. (2014). Estimating and interpreting latent variable interactions: A tutorial for applying the latent moderated structural equations method. *International Journal of Behavioral Development*, *39*(1), 87–96. https://doi.org/10.1177/0165025414552301

Rothbart, M. K., Ahadi, S. A., Hershey, K. L., & Fisher, P. (2001). Investigations of temperament at three to seven years: The Children’s Behavior Questionnaire. *Child Development*, *72*(5), 1394–1408. https://doi.org/https://doi.org/10.1111/1467-8624.00355

Schriber, R. A., & Guyer, A. E. (2016). Adolescent neurobiological susceptibility to social context. *Developmental Cognitive Neuroscience*, *19*, 1–18. https://doi.org/https://doi.org/10.1016/j.dcn.2015.12.009

Simonds, J., Kieras, J. E., Rueda, M. R., & Rothbart, M. K. (2007). Effortful control, executive attention, and emotional regulation in 7–10-year-old children. *Cognitive Development*, *22*(4), 474–488. https://doi.org/10.1016/j.cogdev.2007.08.009

Slagt, M., Dubas, J. S., Deković, M., & van Aken, M. A. G. (2016). Differences in sensitivity to parenting depending on child temperament: A meta-analysis. *Psychological Bulletin*, *142*(10), 1068. https://doi.org/https://doi.org/10.1037/bul0000061

Slagt, M., Dubas, J. S., van Aken, M. A. G., Ellis, B. J., & Deković, M. (2018). Sensory processing sensitivity as a marker of differential susceptibility to parenting. *Developmental Psychology*, *54*(3), 543. https://doi.org/https://doi.org/10.1037/dev0000431

Stright, A. D., Gallagher, K. C., & Kelley, K. (2008). Infant Temperament Moderates Relations Between Maternal Parenting in Early Childhood and Children’s Adjustment in First Grade. *Child Development*, *79*(1), 186–200. https://doi.org/https://doi.org/10.1111/j.1467-8624.2007.01119.x

van de Groep, I. H., Bos, M. G. N., Jansen, L. M. C., Achterberg, M., Popma, A., & Crone, E. A. (2021). Overlapping and distinct neural correlates of self-evaluations and self-regulation from the perspective of self and others. *Neuropsychologia*, *161*, 108000. <https://doi.org/https://doi.org/10.1016/j.neuropsychologia.2021.108000>

Weeland, J., van den Akker, A., Slagt, M., & Putnam, S. (2017). Perception is key? Does perceptual sensitivity and parenting behavior predict children’s reactivity to others’ emotions? *Journal of Experimental Child Psychology*, *163*, 53–68. https://doi.org/https://doi.org/10.1016/j.jecp.2017.06.012

**Supplementary Tables**

**Table S1.** Number of participants with available data of SNAT and Etch-a-sketch measures on 0, 1, 2 and 3 time points.

|  | Number of included time points | | | |
| --- | --- | --- | --- | --- |
|  | 0 time points (n) | 1 time point (n) | 2 time points (n) | 3 time points (n) |
| SNAT: behavior |  | 56 | 130 | 328 |
| SNAT: DLPFC activation | 42 | 126 | 194 | 152 |
| Etch-a-sketch |  | 20 | 63 | 431 |

**Table S2**. Correlations between the responses to feedback, parental sensitivity and temperament measures.

|  |  | 1 | 2 | 3 | 4 | 5 | 6 | 7 | 8 | 9 | 10 | 11 | 12 |
| --- | --- | --- | --- | --- | --- | --- | --- | --- | --- | --- | --- | --- | --- |
| 1. SNAT duration  Δ neg-pos T1 | *r* | - |  |  |  |  |  |  |  |  |  |  |  |
|  | *p* | - |  |  |  |  |  |  |  |  |  |  |  |
| 2. SNAT duration  Δ neg-pos T3 | *r* | **0.25** | - |  |  |  |  |  |  |  |  |  |  |
|  | *p* | <.001 | - |  |  |  |  |  |  |  |  |  |  |
| 3. SNAT duration  Δ neg-pos T5 | *r* | 0.09 | **0.26** | - |  |  |  |  |  |  |  |  |  |
|  | *p* | .094 | <.001 | - |  |  |  |  |  |  |  |  |  |
| 4. DLPFC activation  Δ pos-neg T1 | *r* | **0.49** | **0.15** | 0.05 | - |  |  |  |  |  |  |  |  |
|  | *p* | <.001 | .007 | .397 | - |  |  |  |  |  |  |  |  |
| 5. DLPFC activation  Δ pos-neg T3 | *r* | -0.01 | **0.34** | 0.04 | 0.02 | - |  |  |  |  |  |  |  |
|  | *p* | .811 | <.001 | .515 | .763 | - |  |  |  |  |  |  |  |
| 6. DLPFC activation  Δ pos-neg T5 | *r* | 0.10 | -0.04 | 0.01 | 0.15 | **0.19** | - |  |  |  |  |  |  |
|  | *p* | .129 | .553 | .887 | .059 | .008 | - |  |  |  |  |  |  |
| 7. Etch parental sensitivity T1 | *r* | 0.09 | 0.02 | -0.02 | **0.10** | **0.12** | 0.00 | - |  |  |  |  |  |
|  | *p* | .055 | .716 | .717 | .043 | .023 | .977 | - |  |  |  |  |  |
| 8. Etch parental sensitivity T2 | *r* | 0.03 | -0.06 | 0.01 | 0.01 | 0.02 | -0.08 | **0.42** | - |  |  |  |  |
|  | *p* | .461 | .217 | .835 | .852 | .738 | .256 | <.001 | - |  |  |  |  |
| 9. Etch parental sensitivity T3 | *r* | 0.09 | 0.04 | 0.05 | 0.08 | 0.04 | 0.01 | **0.44** | **0.42** | - |  |  |  |
|  | *p* | .056 | .450 | .376 | .161 | .410 | .919 | <.001 | <.001 | - |  |  |  |
| 10. TMCQ perceptual sensitivity PP | *r* | -0.01 | -0.02 | -0.02 | -0.02 | 0.00 | 0.04 | 0.00 | 0.07 | -0.04 | - |  |  |
|  | *p* | .779 | .618 | .699 | .677 | .994 | .591 | .964 | .123 | .441 | - |  |  |
| 11. TMCQ fear - discomfort child | *r* | **-0.12** | -0.01 | 0.01 | -0.06 | 0.01 | 0.11 | -0.01 | -0.01 | 0.07 | 0.08 | - |  |
|  | *p* | .008 | .907 | .912 | .233 | .918 | .115 | .814 | .835 | .121 | .110 | - |  |
| 12. SNAT duration negative T5 | *r* | -0.01 | **0.18** | **0.85** | -0.03 | 0.05 | -0.08 | -0.06 | -0.01 | -0.01 | 0.00 | 0.00 | - |
|  | *p* | .908 | .001 | <.001 | .647 | .417 | .236 | .244 | .892 | .907 | .951 | .950 | - |
| 13. SNAT duration positive T5 | *r* | **-0.18** | **-0.12** | **-0.22** | **-0.15** | 0.01 | **-0.17** | -0.08 | -0.04 | -0.10 | 0.03 | -0.02 | **0.34** |
|  | *p* | .001 | .030 | <.001 | .015 | .817 | .012 | .129 | .514 | .072 | .601 | .773 | <.001 |

*Note.* Bold numbers indicate significant correlations, *p* < .05.**Table S3**. Model fit and test values for the models with intercept of parental sensitivity as predictor for responses to social feedback at T5 (i.e., noise blast duration and DLPFC activation), controlled for VIPP and sex.

| *Parental sensitivity (I) 🡪 Responses to feedback* | **Model fit** | | | |  | **Test values** | | |
| --- | --- | --- | --- | --- | --- | --- | --- | --- |
|  | Χ^2^ | CFI | TLI | RMSEA [95%CI] |  | *β* | *p* | 95%CI |
| ***Noise blast duration T5*** |  |  |  |  |  |  |  |  |
| negative - positive | *Χ^2^*(11) = 13.78, *p* = .245 | 0.988 | 0.984 | 0.023 [0.00, 0.06] |  | 0.014 | .852 | [-0.131, 0.158] |
| negative - neutral | *Χ^2^*(11) = 12.93, *p* = .298 | 0.991 | 0.989 | 0.019 [0.00, 0.05] |  | 0.070 | .390 | [-0.089, 0.229] |
| negative | *Χ^2^*(11) = 13.60, *p* = .996 | 0.988 | 0.985 | 0.022 [0.00, 0.06] |  | -0.046 | .539 | [-0.192, 0.100] |
| positive | *Χ^2^*(11) = 14.08, *p* = .970 | 0.986 | 0.983 | 0.024 [0.00, 0.06] |  | -0.111 | .095 | [-0.241, 0.019] |
| ***DLPFC activation T5*** |  |  |  |  |  |  |  |  |
| positive – negative | *Χ^2^*(11) = 14.16, *p* = .224 | 0.986 | 0.982 | 0.024 [0.00, 0.06] |  | -0.030 | .726 | [-0.199, 0.139] |
| neutral - negative | *Χ^2^*(11) = 14.70, *p* = .197 | 0.983 | 0.979 | 0.026 [0.00, 0.06] |  | 0.013 | .882 | [-0.156, 0.182] |

**Table S4**. Model fit indices for models testing main effects of parental sensitivity and temperament on responses to social feedback with and without the addition of sex and VIPP.

|  | **Model fit null model (only main effects)** | | | |
| --- | --- | --- | --- | --- |
|  | Χ^2^ | CFI | TLI | RMSEA [90% CI] |
| **Noise blast duration T5** |  |  |  |  |
| ***Negative - Positive*** |  |  |  |  |
| Perceptual sensitivity | Χ^2^(7) = 16.84, *p* = .019 | .956 | .937 | 0.052 [0.020, 0.085] |
| With sex & VIPP | Χ^2^(15) = 30.08, *p* = .012 | .934 | .921 | 0.045 [0.021, 0.068] |
| Fear and discomfort | Χ^2^(7) = 14.68, *p* = .040 | .967 | .952 | 0.046 [0.009, 0.079] |
| With sex & VIPP | Χ^2^(15) = 66.05, *p* < .001 | .778 | .733 | 0.083 [0.063, 0.104] |
| ***Negative – Neutral*** |  |  |  |  |
| Perceptual sensitivity | Χ^2^(7) = 15.86, *p* = .026 | 0.961 | 0.944 | 0.050 [0.016, 0.082] |
| With sex & VIPP | Χ^2^(15) = 29.00, *p* = .016 | 0.938 | 0.926 | 0.043 [0.018, 0.067] |
| Fear and discomfort | Χ^2^(7) = 13.83, *p* = .054 | 0.969 | 0.956 | 0.044 [0.000, 0.077] |
| With sex & VIPP | Χ^2^(15) = 62.26, *p* < .001 | 0.773 | 0.728 | 0.082 [0.062, 0.103] |
| ***Negative*** |  |  |  |  |
| Perceptual sensitivity | Χ^2^(7) = 16.42, *p* = .022 | .958 | .940 | 0.051 [0.018, 0.084] |
| With sex & VIPP | Χ^2^(15) = 29.67, *p* = .013 | .936 | .923 | 0.044 [0.020, 0.068] |
| Fear and discomfort | Χ^2^(7) = 14.44, *p* = .044 | .967 | .953 | 0.045 [0.007. 0.079] |
| With sex & VIPP | Χ^2^(15) = 65.41, *p* < .001 | .779 | .735 | 0.082 [0.063, 0.103] |
| ***Positive*** |  |  |  |  |
| Perceptual sensitivity | Χ^2^(7) = 17.38, *p* = .015 | 0.956 | 0.938 | 0.054 [0.022, 0.086] |
| With sex & VIPP | Χ^2^(15) = 30.50, *p* = .010 | 0.933 | 0.920 | 0.046 [0.022, 0.069] |
| Fear and discomfort | Χ^2^(7) = 15.12, *p* = .035 | 0.965 | 0.950 | 0.047 [0.012, 0.081] |
| With sex & VIPP | Χ^2^(15) = 66.89, *p* < .001 | 0.773 | 0.727 | 0.084 [0.064, 0.105] |
|  |  |  |  |  |
| **DLPFC activation T5** |  |  |  |  |
| ***Positive – Negative*** |  |  |  |  |
| Perceptual sensitivity | Χ^2^(7) = 17.47, *p* = .015 | 0.955 | 0.936 | 0.054 [0.022, 0.086] |
| With sex & VIPP | Χ^2^(15) = 30.61, *p* = .001 | 0.932 | 0.918 | 0.046 [0.022, 0.069] |
| Fear and discomfort | Χ^2^(7) = 14.96, *p* = .037 | 0.965 | 0.950 | 0.047 [0.011, 0.080] |
| With sex & VIPP | Χ^2^(15) = 66.35, *p* < .001 | 0.772 | 0.726 | 0.083 [0.063, 0.104] |
| ***Neutral – Negative*** |  |  |  |  |
| Perceptual sensivity | Χ^2^(7) = 17.57, *p* = .014 | 0.954 | 0.935 | 0.054 [0.023, 0.086] |
| With sex & VIPP | Χ^2^(15) = 31.07, *p* = .009 | 0.930 | 0.916 | 0.047 [0.023, 0.070] |
| Fear and discomfort | Χ^2^(7) = 15.31, *p* = .032 | 0.964 | 0.948 | 0.048 [0.013, 0.081] |
| With sex & VIPP | Χ^2^(15) = 67.49, *p* < .001 | 0.769 | 0.722 | 0.084 [0.064, 0.105] |

**Table S5.** Model fit statistics comparing models with and without interaction variable.

|  | **Model fit null model (main effects)** | | | **Model fit interaction (and main effects)** | | | **Model fit comparison** |
| --- | --- | --- | --- | --- | --- | --- | --- |
|  | log likelihood | No. of parameters | MLR scaling factor | log likelihood | No. of parameters | MLR scaling factor | Δ null model – interaction model |
| **Noise blast duration T5** |  |  |  |  |  |  |  |
| ***Negative – Positive*** |  |  |  |  |  |  |  |
| Perceptual sensitivity | -3172.421 | 13 | 1.1416 | -2919.188 | 14 | 1.1815 | Χ^2^(1) = 297.89, *p* <.001 |
| Fear and discomfort | -3170.558 | 13 | 1.0781 | -3125.473 | 14 | 1.0640 | Χ^2^(1) = 102.38, *p* <.001 |
| ***Negative – Neutral*** |  |  |  |  |  |  |  |
| Perceptual sensitivity | -3034.060 | 13 | 1.1849 | -2790.365 | 14 | 1.2007 | Χ^2^(1) = 346.85, *p* <.001 |
| Fear and discomfort | -3032.694 | 13 | 1.1473 | -2987.427 | 14 | 1.1724 | Χ^2^(1) = 60.41, *p* <.001 |
| ***Negative*** |  |  |  |  |  |  |  |
| Perceptual sensitivity | -3183.779 | 13 | 1.1199 | -2928.062 | 14 | 1.1264 | Χ^2^(1) = 511.43, *p* <.001 |
| Fear and discomfort | -3181.849 | 13 | 1.0889 | -3136.707 | 14 | 1.0832 | Χ^2^(1) = 89.47, *p* <.001 |
| ***Positive*** |  |  |  |  |  |  |  |
| Perceptual sensitivity | -2976.993 | 13 | 1.2218 | -2734.594 | 14 | 1.2175 | Χ^2^(1) = 417.35, *p* <.001 |
| Fear and discomfort | -2975.158 | 13 | 1.1866 | -2929.916 | 14 | 1.1858 | Χ^2^(1) = 76.98, *p* <.001 |
|  |  |  |  |  |  |  |  |
| **DLPFC activation T5** |  |  |  |  |  |  |  |
| ***Positive – Negative*** |  |  |  |  |  |  |  |
| Perceptual sensitivity | -3371.987 | 13 | 1.1971 | -3110.000 | 14 | 1.1796 | Χ^2^(1) = 550.34, *p* <.001 |
| Fear and discomfort | -3368.900 | 13 | 1.1771 | -3323.737 | 14 | 1.1459 | Χ^2^(1) = 122.01, *p* <.001 |
| ***Neutral – Negative*** |  |  |  |  |  |  |  |
| Perceptual sensitivity | -3253.778 | 13 | 1.2119 | -3003.526 | 14 | 1.1888 | Χ^2^(1) = 563.32, *p* <.001 |
| Fear and discomfort | -3251.068 | 13 | 1.1713 | -3205.498 | 14 | 1.2076 | Χ^2^(1) = 54.27, *p* <.001 |

*Note.* Model fit was compared with log-likelihood tests with MLR estimators, using the following formula: -2 [(loglikelihood null model – loglikelihood interaction model)] / [(no. of parameters null model * MLR scaling factor – no. of parameters interaction model * MLR scaling factor) / (no. of parameters null model – no. of parameters interaction model)].

|  | **Parental sensitivity (I) 🡪**  **responses to feedback** | | |  | **Temperament 🡪**  **responses to feedback** | | |  | **Parental sensitivity (I) * temperament 🡪**  **responses to feedback** | | |
| --- | --- | --- | --- | --- | --- | --- | --- | --- | --- | --- | --- |
|  | *β* | *p* | 95%CI |  | *β* | *p* | 95%CI |  | *β* | *p* | 95%CI |
| **Noise blast T5** |  |  |  |  |  |  |  |  |  |  |  |
| ***Negative – Positive*** |  |  |  |  |  |  |  |  |  |  |  |
| Moderator: perceptual sensitivity | 0.021 | .790 | [-0.135, 0.177] |  | 0.059 | .899 | [-0.855, 0.974] |  | -0.018 | .863 | [-0.221, 0.186] |
| Moderator: fear and discomfort | 0.013 | .861 | [-0.133, 0.159] |  | -0.014 | .961 | [-0.571, 0.543] |  | 0.004 | .946 | [-0.121, 0.130] |
| ***Negative – Neutral*** |  |  |  |  |  |  |  |  |  |  |  |
| Moderator: perceptual sensitivity | 0.062 | .485 | [-0.112, 0.236] |  | 0.462 | .236 | [-0.302, 1.226] |  | -0.086 | .342 | [-0.264, 0.092] |
| Moderator: fear and discomfort | 0.063 | .447 | [-0.099, 0.224] |  | -0.176 | .653 | [-0.943, 0.591] |  | 0.045 | .621 | [-0.133, 0.223] |
| ***Negative feedback*** |  |  |  |  |  |  |  |  |  |  |  |
| Moderator: perceptual sensitivity | -0.038 | .621 | [-0.186, 0.111] |  | 0.082 | .830 | [-0.663, 0.826] |  | -0.019 | .820 | [-0.180, 0.142] |
| Moderator: fear and discomfort | -0.051 | .506 | [-0.201, 0.099] |  | -0.109 | .741 | [-0.753, 0.536] |  | 0.024 | .735 | [-0.114, 0.162] |
| ***Positive feedback*** |  |  |  |  |  |  |  |  |  |  |  |
| Moderator: perceptual sensitivity | -0.113 | .114 | [-0.254, 0.027] |  | 0.108 | .775 | [-0.633, 0.849] |  | -0.017 | .835 | [-0.178, 0.144] |
| Moderator: fear and discomfort | -0.121 | .092 | [-0.262, 0.020] |  | -0.195 | .598 | [-0.920, 0.530] |  | 0.041 | .602 | [-0.113, 0.194] |
|  |  |  |  |  |  |  |  |  |  |  |  |
| **DLPFC activation T5** |  |  |  |  |  |  |  |  |  |  |  |
| ***Positive – Negative*** |  |  |  |  |  |  |  |  |  |  |  |
| Moderator: perceptual sensitivity | -0.042 | .651 | [-0.222, 0.139] |  | -0.009 | .983 | [-0.818, 0.801] |  | 0.009 | .919 | [-0.167, 0.185] |
| Moderator: fear and discomfort | -0.028 | .742 | [-0.198, 0.141] |  | 0.273 | .417 | [-0.386, 0.932] |  | -0.028 | .642 | [-0.186, 0.114] |
| ***Neutral – Negative*** |  |  |  |  |  |  |  |  |  |  |  |
| Moderator: perceptual sensitivity | 0.034 | .705 | [-0.144, 0.213] |  | -0.113 | .775 | [-0.890, 0.664] |  | 0.037 | .655 | [-0.126, 0.201] |
| Moderator: fear and discomfort | 0.028 | .742 | [-0.138, 0.193] |  | 0.507 | .321 | [-0.495, 1.509] |  | -0.089 | .442 | [-0.317, 0.138] |
|  |  |  |  |  |  |  |  |  |  |  |  |
| **Noise blast Intercept: positive** |  |  |  |  |  |  |  |  |  |  |  |
| Moderator: perceptual sensitivity | -0.226 | .010 | [-0.399, -0.054] |  | -0.019 | .946 | [-0.563, 0.526] |  | -0.001 | .983 | [-0.114, 0.112] |
| Moderator: fear and discomfort | -0.242 | .005 | [-0.409, -0.074] |  | -0.304 | .267 | [-0.840, 0.233] |  | 0.091 | .108 | [-0.020, 0.203] |

**Table S6.** Statistical test values for the moderation models with main and interaction effects of parental sensitivity and temperament on responses to social feedback.

**Table S7.** MNI coordinates for local maxima activation for the whole-brain contrasts PositiveNoise > NegativeNoise and NegativeNoise > PositiveNoise at T1, T3 and T5.

|  |  |  |  | **MNI coordinates** | | |
| --- | --- | --- | --- | --- | --- | --- |
| **Anatomical region** | **Voxels** | ***p*FWEcc** | ***T*** | ***x*** | ***y*** | **Z** |
| ***T1: PositiveNoise > NegativeNoise*** | | | | | | |
| Right inferior parietal lobule | 2799 | < .001 | 7.52 | 50 | -44 | 52 |
|  |  |  | 6.29 | 50 | -58 | 46 |
| Right angular gyrus |  |  | 5.74 | 36 | -68 | 52 |
| Left lingual gyrus | 2330 | < .001 | 6.91 | -8 | -80 | 2 |
| Left superior occipital gyrus |  |  | 6.66 | -16 | -88 | 16 |
| Right calcarine gyrus |  |  | 6.54 | 12 | -80 | 4 |
| Right precentral gyrus | 1829 | < .001 | 6.81 | 46 | -10 | 30 |
| Right insula lobe |  |  | 5.44 | 36 | -14 | 16 |
| Right heschls gyrus |  |  | 5.06 | 38 | -26 | 14 |
| Right middle frontal gyrus | 4155 | < .001 | 6.50 | 44 | 40 | 16 |
| Left superior medial gyrus |  |  | 5.72 | -6 | 38 | 32 |
| Right inferior frontal gyrus |  |  | 5.70 | 48 | 20 | 34 |
| Left middle frontal gyrus | 870 | .001 | 6.22 | -42 | 46 | 8 |
|  |  |  | 4.98 | -44 | 38 | 16 |
| Left precentral gyrus |  |  | 4.78 | -50 | 8 | 40 |
| Left inferior parietal lobule | 837 | .001 | 6.14 | -44 | -56 | 52 |
| Left supramarginal gyrus |  |  | 5.41 | -58 | -52 | 34 |
| Left angular gyrus |  |  | 4.16 | -48 | -62 | 36 |
| Left postcentral gyrus | 494 | .011 | 5.46 | -42 | -18 | 34 |
|  |  |  | 5.24 | -54 | -12 | 30 |
|  |  |  | 5.01 | -60 | -6 | 24 |
|  |  |  |  |  |  |  |
| ***T1: NegativeNoise > PositiveNoise*** | | | | | | |
| Left postcentral gyrus | 344 | 0.034 | 5.33 | -36 | -22 | 52 |
|  |  |  |  |  |  |  |
| ***T1: NeutralNoise > NegativeNoise*** | |  |  |  |  |  |
| Right superior frontal gyrus | 10653 | < .001 | 7.89 | 28 | 0 | 54 |
| Right angular gyrus |  |  | 7.27 | 32 | -66 | 46 |
| Right inferior parietal lobule |  |  | 7.23 | 40 | -46 | 44 |
| Right middle frontal gyrus | 887 | < .001 | 7.15 | 46 | 40 | 20 |
|  |  |  | 5.55 | 44 | 44 | 8 |
|  |  |  | 5.22 | 40 | 32 | 34 |
| Left superior parietal lobule |  |  | 5.75 | -30 | -66 | 54 |
| Left inferior parietal lobule |  |  | 5.54 | -42 | -48 | 50 |
|  |  |  | 5.46 | -36 | -46 | 38 |
| Left middle frontal gyrus |  |  | 5.24 | -26 | 0 | 56 |
| Left precentral gyrus |  |  | 5.05 | -48 | 4 | 36 |
| Left postcentral gyrus |  |  | 4.77 | -44 | -18 | 36 |
|  |  |  | 4.75 | -54 | -12 | 30 |
|  |  |  |  |  |  |  |
| ***T1: NegativeNoise > NeutralNoise*** | |  |  |  |  |  |
| n.s. | |  |  |  |  |  |
|  |  |  |  |  |  |  |
| ***T3: PositiveNoise > NegativeNoise*** | | | | | | |
| Left calcarine gyrus | 42703 | < .001 | 13.28 | -10 | -86 | 2 |
| Left lingual gyrus |  |  | 10.71 | -10 | -74 | -4 |
| Right calcarine gyrus |  |  | 10.31 | 20 | -90 | 4 |
| Left middle temporal gyrus | 221 | .022 | 4.42 | -58 | -36 | -8 |
|  |  |  | 3.90 | -56 | -50 | -8 |
|  |  |  | 3.65 | -62 | -26 | -8 |
|  |  |  |  |  |  |  |
| ***T3: NegativeNoise > PositiveNoise*** | | | | | | |
| Left postcentral gyrus | 767 | < .001 | 8.04 | -32 | -26 | 48 |
| Right precuneus | 375 | .002 | 7.51 | 22 | -44 | 16 |
| Right calcarine gyrus |  |  | 4.39 | 30 | -52 | 8 |
| Right fusiform gyrus |  |  | 3.19 | 34 | -48 | -2 |
| Left supplementary motor area | 923 | < .001 | 6.91 | -6 | 0 | 52 |
| Right supplementary motor area |  |  | 5.93 | 12 | 6 | 46 |
| Left middle cingulate cortex |  |  | 5.66 | -10 | -24 | 46 |
| Right middle temporal gyrus | 181 | .045 | 6.67 | 42 | -64 | 2 |
| Left precuneus | 232 | .018 | 6.14 | -18 | -44 | 16 |
|  |  |  |  |  |  |  |
| ***T3: NeutralNoise > NegativeNoise*** | |  |  |  |  |  |
| Right superior parietal lobule | 12915 | < .001 | 9.08 | 28 | -62 | 54 |
| Right supramarginal gyrus |  |  | 8.51 | 44 | -36 | 44 |
| Right middle frontal gyrus |  |  | 8.12 | 44 | 40 | 22 |
| Left inferior parietal lobule | 2096 | < .001 | 6.69 | -42 | -48 | 46 |
| Left superior parietal lobule |  |  | 6.38 | -18 | -70 | 52 |
|  |  |  | 6.29 | -28 | -66 | 54 |
| Right inferior temporal gyrus | 476 | < .001 | 6.17 | 52 | -50 | -14 |
|  |  |  | 5.91 | 50 | -58 | -14 |
| Right inferior occipital gyrus |  |  | 3.22 | 36 | -70 | -10 |
| Right superior medial gyrus | 729 | < .001 | 6.07 | 4 | 24 | 44 |
| Right middle cingulate cortex |  |  | 4.04 | 2 | 12 | 38 |
| Right SMA |  |  | 3.44 | 2 | 14 | 58 |
| Left inferior frontal gyrus | 1963 | < .001 | 6.02 | -44 | 42 | 0 |
| Left postcentral gyrus |  |  | 5.81 | -40 | -16 | 36 |
|  |  |  | 5.46 | -52 | -8 | 28 |
| Left precentral gyrus | 323 | .004 | 5.71 | -30 | -4 | 60 |
| Left middle occipital gyrus | 462 | < .001 | 4.83 | -26 | -88 | 6 |
|  |  |  | 4.59 | -22 | -88 | 14 |
| Left superior occipital gyrus |  |  | 4.56 | -14 | -88 | 4 |
| Right caudate nucleus | 300 | .006 | 4.57 | 22 | -6 | 24 |
| Right middle cingulate cortex |  |  | 4.26 | 10 | 8 | 26 |
|  |  |  | 3.84 | 6 | -2 | 28 |
|  |  |  |  |  |  |  |
| ***T3: NegativeNoise > NeutralNoise*** | |  |  |  |  |  |
| Right middle temporal gyrus | 559 | < .001 | 5.78 | 58 | -58 | 18 |
|  |  |  | 5.45 | 60 | -50 | 26 |
| Right supramarginal gyrus |  |  | 4.98 | 60 | -50 | 14 |
| Left postcentral gyrus | 331 | .004 | 5.65 | -34 | -26 | 48 |
| Left middle temporal gyrus | 893 | < .001 | 5.29 | -46 | -60 | 20 |
| Left middle occipital gyrus |  |  | 5.02 | -42 | -74 | 34 |
| Left angular gyrus |  |  | 4.90 | -50 | -70 | 24 |
| Right middle temporal gyrus | 427 | <.001 | 5.14 | 56 | -14 | -14 |
|  |  |  | 4.69 | 52 | -4 | -20 |
| Left middle temporal gyrus | 278 | .009 | 5.13 | -56 | -16 | -14 |
|  |  |  | 4.98 | -56 | -8 | -16 |
| Left middle cingulate cortex | 669 | < .001 | 4.46 | -2 | -46 | 34 |
| Right precuneus |  |  | 4.42 | 12 | -52 | 32 |
| Left precuneus |  |  | 4.21 | -4 | -56 | 22 |
|  |  |  |  |  |  |  |
| ***T5: PositiveNoise > NegativeNoise*** | | | | | | |
| Left calcarine gyrus | 14901 | < .001 | 10.35 | -8 | -82 | 0 |
| Left cerebellum |  |  | 10.28 | -10 | -74 | -12 |
| Left calcarine gyrus |  |  | 8.92 | -10 | -88 | 8 |
| Right precentral gyrus | 1531 | < .001 | 5.55 | 40 | -12 | 36 |
| Right rolandic operculum |  |  | 4.65 | 60 | -2 | 8 |
|  |  |  | 4.62 | 58 | 6 | 2 |
| Right middle frontal gyrus | 469 | .004 | 5.41 | 46 | 44 | 6 |
|  |  |  | 4.64 | 44 | 46 | 16 |
|  |  |  | 4.37 | 40 | 52 | 2 |
| Left postcentral gyrus | 697 | .001 | 4.88 | -44 | -14 | 32 |
|  |  |  | 4.69 | -52 | -14 | 26 |
|  |  |  | 4.51 | -58 | -12 | 34 |
|  |  |  |  |  |  |  |
| ***T5: NegativeNoise > PositiveNoise*** | | | | | | |
| n.s. |  |  |  |  |  |  |
|  |  |  |  |  |  |  |
| ***T5: NeutralNoise > NegativeNoise*** | |  |  |  |  |  |
| Left lingual gyrus | 8126 | < .001 | 5.45 | -10 | -58 | -2 |
| Left calcarine gyrus |  |  | 5.17 | -10 | -88 | -4 |
| Right calcarine gyrus |  |  | 5.09 | 12 | -90 | 4 |
|  |  |  |  |  |  |  |
| ***T5: NegativeNoise > NeutralNoise*** | |  |  |  |  |  |
| n.s. |  |  |  |  |  |  |
|  |  |  |  |  |  |  |

|  | **Total sample** | |  | **Sample A**  **(half sample)** | |  | **Sample B**  **(half sample)** | |
| --- | --- | --- | --- | --- | --- | --- | --- | --- |
|  | *r*  [95% CI] | *p* |  | *r*  [95% CI] | *p* |  | *r*  [95% CI] | *p* |
| ***Difference score negative – positive*** |  |  |  |  |  |  |  |  |
| SNAT noise blast – DLPFC at T1 | 0.49  [0.40,0.56] | <.001 |  | 0.51  [0.39,0.61] | <.001 |  | 0.48  [0.36,0.58] | <.001 |
| SNAT noise blast – DLPFC at T3 | 0.34  [0.24,0.43] | <.001 |  | 0.40  [0.27,0.51] | <.001 |  | 0.27  [0.13,0.40] | <.001 |
| SNAT noise blast – DLPFC at T5 | 0.01  [-0.13,0.14] | .887 |  | -0.10  [-0.28,0.08] | .283 |  | 0.13  [-0.06,0.31] | .185 |
| ***Negative / neutral / positive at T5*** |  |  |  |  |  |  |  |  |
| SNAT noise blast negative – DLPFC at T5 | -0.08  [-0.21,0.05] | .236 |  | -0.19  [-0.36,-0.01] | .042 |  | 0.04  [-0.15,0.23] | .658 |
| SNAT noise blast neutral – DLPFC at T5 | -0.15  [-0.28,-0.02] | .022 |  | -0.24  [-0.41,-0.06] | .010 |  | -0.07  [-0.26,0.12] | .460* |
| SNAT noise blast positive – DLPFC at T5 | -0.17  [-0.29,-0.04] | .012 |  | -0.18  [-0.35,0.01] | .057* |  | -0.16  [-0.34,0.03] | .101* |
| ***Difference score negative – neutral*** |  |  |  |  |  |  |  |  |
| SNAT noise blast – DLPFC at T1 | 0.48  [0.40,0.55] | <.001 |  | 0.50  [0.39,0.60] | <.001 |  | 0.45  [0.33,0.56] | <.001 |
| SNAT noise blast – DLPFC at T3 | 0.36  [0.27,0.45] | <.001 |  | 0.32  [0.18,0.44] | <.001 |  | 0.40  [0.27,0.51] | <.001 |
| SNAT noise blast – DLPFC at T5 | 0.15  [0.02,0.27] | .029 |  | 0.12  [-0.07,0.30] | .208* |  | 0.18  [-0.01,0.35] | .069* |

**Table S8.** Correlations between noise blast duration and DLPFC activation in the total sample and split-half samples A and B with one child per twin pair in each sample.

*Note.* Confidence intervals of effects in total samples and split-half samples all overlapped. * = results were no longer significant in split-half sample.

| *Development* | **Total sample**  **(random effect FamilyID)** | | | |  | **Sample A (half sample)** | | | |  | **Sample B (half sample)** | | | |
| --- | --- | --- | --- | --- | --- | --- | --- | --- | --- | --- | --- | --- | --- | --- |
|  | *F* | *df* | *p* | η^2^_p_ |  | *F* | *df* | *p* | η^2^_p_ |  | *F* | *df* | *p* | η^2^_p_ |
| ***SNAT noise duration*** | | |  |  |  |  |  |  |  |  |  |  |  |  |
| Feedback | 1655.49 | 2,3335.2 | <.001 | 0.50 |  | 928.62 | 2,1673.66 | <.001 | 0.53 |  | 741.06 | 2,1661.51 | <.001 | 0.47 |
| Wave | 265.76 | 2,3461.4 | <.001 | 0.13 |  | 149.58 | 2,1756.89 | <.001 | 0.15 |  | 117.97 | 2,1732.95 | <.001 | 0.12 |
| Feedback*Wave | 19.15 | 4,3335.2 | <.001 | 0.02 |  | 9.48 | 4,1673.71 | <.001 | 0.02 |  | 10.35 | 4,1661.50 | <.001 | 0.02 |
| ***SNAT negative – positive*** | | |  |  |  |  |  |  |  |  |  |  |  |  |
| Wave | 32.79 | 2,833.77 | <.001 | 0.07 |  | 16.25 | 2,427.19 | <.001 | 0.07 |  | 17.19 | 2,414.29 | <.001 | 0.08 |
| ***SNAT negative - neutral*** | | |  |  |  |  |  |  |  |  |  |  |  |  |
| Wave | 14.15 | 2,842.69 | <.001 | 0.03 |  | 8.56 | 2,430.94 | <.001 | 0.04 |  | 6.99 | 2,423.71 | .001 | 0.03 |
| ***DLPFC activation*** | |  |  |  |  |  |  |  |  |  |  |  |  |  |
| Feedback | 84.69 | 2,2403.54 | <.001 | 0.07 |  | 41.37 | 2,1215.89 | <.001 | 0.06 |  | 44.45 | 2,1186.18 | <.001 | 0.07 |
| Wave | 27.32 | 2,2745.10 | <.001 | 0.02 |  | 17.64 | 2.1406.68 | <.001 | 0.02 |  | 11.49 | 2,1373.25 | <.001 | 0.02 |
| Feedback*Wave | 2.68 | 4,2403.54 | .030 | 0.004 |  | 1.64 | 4,1215.89 | .163* | 0.005 |  | 1.36 | 4,1186.18 | .248* | 0.005 |

**Table S9.** Results of the linear mixed models on the development of noise blast durations and DLPFC activation in the total sample and split-half samples A and B with one child per twin pair in each sample.

* = results were no longer significant in split-half sample.

| *Parental sensitivity (I) 🡪 Responses to feedback* | **Total sample**  **(random effect FamilyID)** | | |  | **Sample A (half sample)** | | |  | **Sample B (half sample)** | | |
| --- | --- | --- | --- | --- | --- | --- | --- | --- | --- | --- | --- |
|  | *β* | *p* | 95%CI |  | *β* | *p* | 95%CI |  | *β* | *p* | 95%CI |
| ***Noise blast duration T5*** | |  |  |  |  |  |  |  |  |  |  |
| negative – positive | 0.014 | .852 | [-0.131, 0.158] |  | 0.075 | .482 | [-0.133, 0.283] |  | -0.039 | .680 | [-0.224, 0.146] |
| negative – neutral | 0.070 | .390 | [-0.089, 0.229] |  | -0.190 | .070 | [-0.015, 0.396] |  | -0.023 | .826 | [-0.227, 0.181] |
| negative | -0.046 | .539 | [-0.192, 0.100] |  | 0.017 | .867 | [-0.187, 0.222] |  | -0.101 | .261 | [-0.278, 0.075] |
| positive | -0.111 | .095 | [-0.241, 0.019] |  | -0.098 | .288 | [-0.280, 0.083] |  | -0.119 | .203 | [-0.302, 0.064] |
| ***DLPFC activation T5*** |  |  |  |  |  |  |  |  |  |  |  |
| positive - negative | -0.030 | .726 | [-0.199, 0.139] |  | -0.014 | .899 | [-0.238, 0.209] |  | -0.054 | .643 | [-0.283, 0.174] |
| neutral – negative | 0.013 | .882 | [-0.156, 0.182] |  | -0.059 | .645 | [-0.307, 0.190] |  | 0.085 | .469 | [-0.146, 0.317] |
| ***Bivariate growth curve model*** | |  |  |  |  |  |  |  |  |  |  |
| Noise blast intercept: positive | -0.223 | .011 | [-0.394, -0.052] |  | -0.123 | .376* | [-0.394, 0.149] |  | -0.294 | .007 | [-.508, 0.080] |

**Table S10.** Statistical test values for the growth curve models on associations between parenting (intercept) and responses to social feedback at T5 in the total sample and split-half samples A and B with one child per twin pair in each sample.

*Note.* Confidence intervals of effects in total sample and split-half samples all overlapped. * = results were no longer significant in split-half sample.

**Supplementary Figures**

**
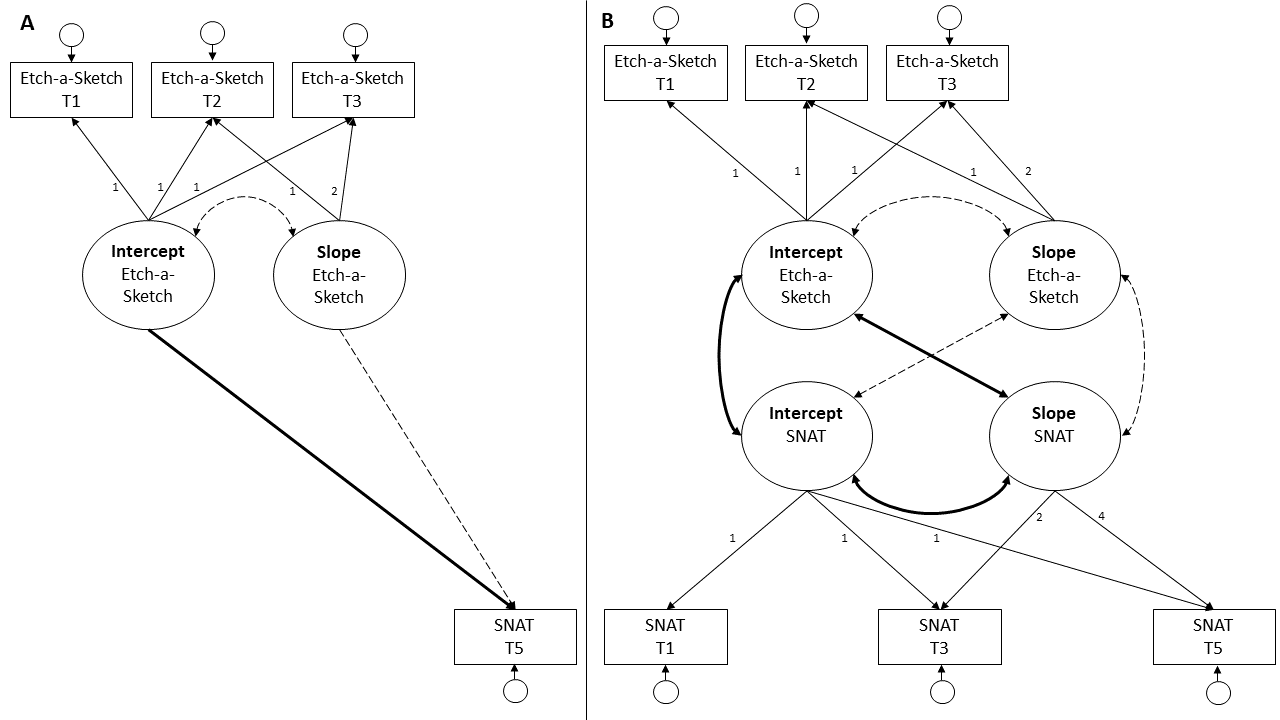
Figure S1.** Path diagram of the latent growth curve models for parental sensitivity (Etch-a-Sketch) and responses to social feedback (SNAT noise blast duration / DLFPC activation). Numbers indicate factor loadings, bold solid lines indicate relations of interest, dotted lines indicate relations that could not be tested because there was no between-subject variance in the slope of Etch-a-Sketch. A) Latent growth curve model for relation between intercept/slope Etch-a-Sketch and SNAT measures (noise blast duration/DLPFC activation) at T5. B) Bivariate latent growth curve model for relation between intercept/slope Etch-a-Sketch and intercept/slope SNAT.


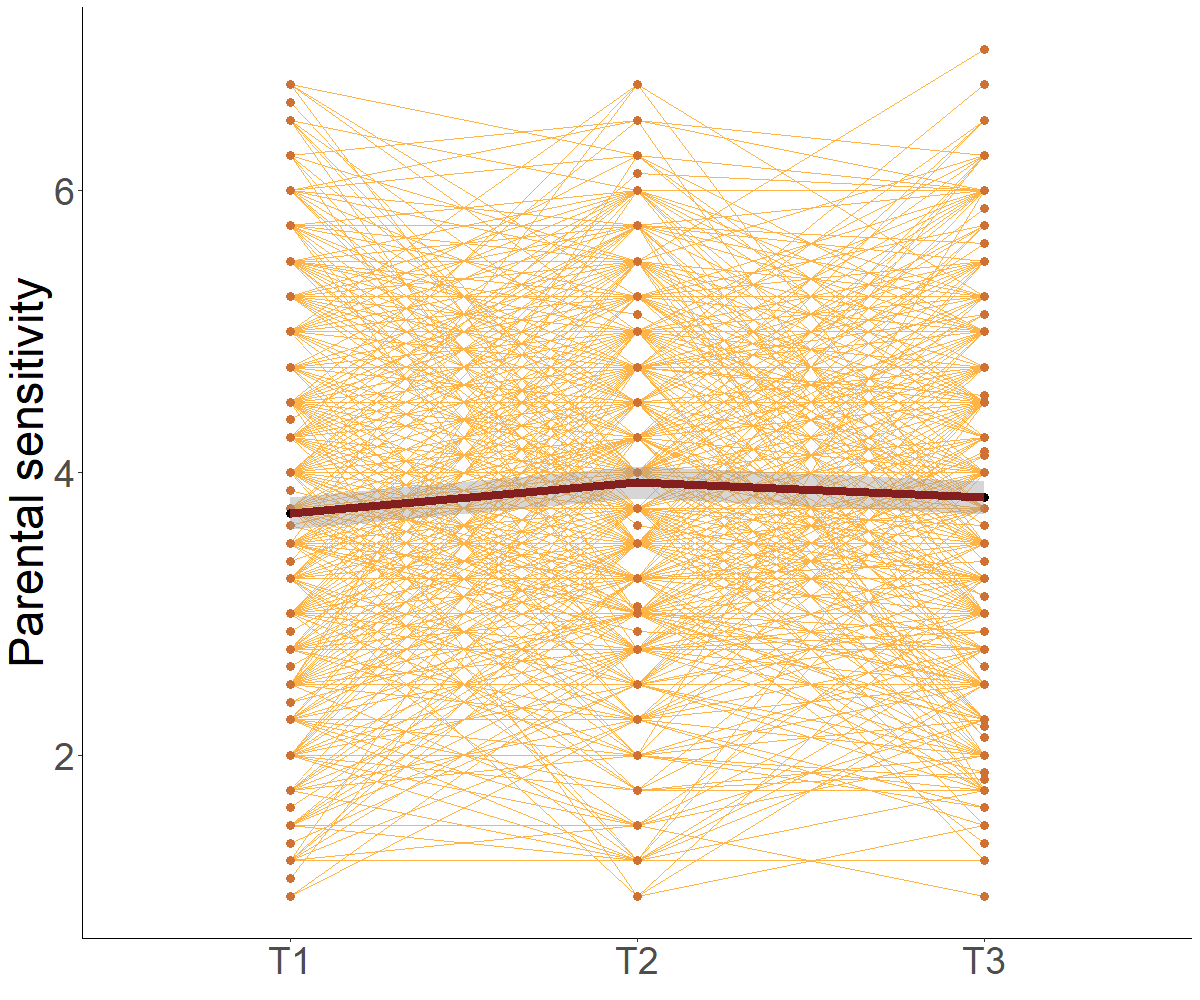


**Figure S2**. Developmental trajectory across T1, T2 and T3 for parental sensitivity scores.
